# How Perspective Directs Outcomes: The Structure and Function of Deficit and Anti-deficit Framing and Their Relevance to Chemistry Education

**DOI:** 10.1021/jacsau.6c00081

**Published:** 2026-04-17

**Authors:** Elizabeth B. Vaughan, Josephine Bicknell, Henry Holleb, Kodinna Anachebe, Tia Kledzik, Nicole M. James

**Affiliations:** Department of Chemistry, 6686 Reed College, Portland, Oregon 97202, United States

**Keywords:** chemistry education, chemistry education research, deficit framing, deficit thinking, anti-deficit, equity, achievement, outcomes

## Abstract

The existence of differential educational outcomes as a function of students’ background is well-established and especially severe in science, technology, engineering, and mathematics (STEM). Differential educational outcomes adversely impact chemistry education and training as well as education and training in other STEM disciplines that employ chemistry. This has motivated intensive efforts to improve chemistry teaching and learning. However, one particularly powerful approach for improving educational outcomes has been largely neglected in postsecondary chemistry education: the ability to recognize and resist the pervasive deficit framed perspectives held by and about chemistry education stakeholders (e.g., students, educators, institutions, etc.). In this integrative systematic literature review, we formulate a comprehensive description of the structure and function of deficit framing and its alternative—anti-deficit framing. In particular: deficit framed perspectives prompt actions and patterns of thought that reduce stakeholder expectations and achievement, while anti-deficit framed perspectives promote higher expectations and improved achievement. However, the pervasive, tacit, and self-reinforcing nature of deficit framing means that anti-deficit perspectives require conscious intentionality and self-reflection to maintain. These findings have implications for chemistry education research and can inform the rational design of solutions for approaching chemistry education and training in ways that maintain rigorous standards and support stakeholders’ achievement.

## Graphical abstract

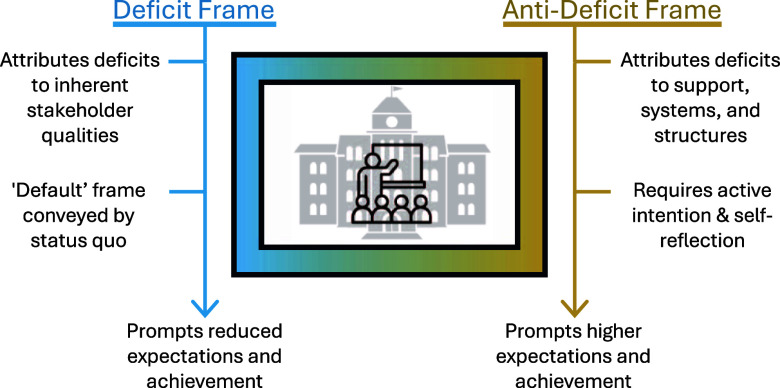

## Introduction

Of all societal institutions, educational systems have some of the most influence on individuals’ socialization, social mobility, knowledge, and skills.^1,2^ Education is a gateway and can provide access to knowledge, skills, and new opportunities. However, educational systems also function as gatekeepers, presenting barriers to accessing knowledge, skills, and opportunities.^3,4^ It is well established that these barriers differentially impact individuals along factors such as race and ethnicity,^5–10^ gender,^8,9,11,12^ socioeconomic status,^8,9,11,13^ sexual orientation,^14^ and disability.^15,16^ This is especially true in chemistry and other STEM fields, which are associated with lower academic grades than other disciplines^17,18^ and severe differential outcomes for students from marginalized backgrounds.^19–25^

Many complex and nuanced factors result in these differential outcomes. One broadly influential factor that is especially relevant is deficit framing. In his seminal work, R. R. Valencia describes deficit-based perspectives as a “victim-blaming” paradigm that attributes students’ school failure to personal qualities that are unavoidable and inherent to who they are biologically, culturally, and/or behaviorally.^26^ Here, we refer to this paradigm in terms of *frames* and *framing*.

Frames guide what one attends to, understands, and thinks about a situation, thereby influencing how one responds.^27,28^ Framing is a dynamic process of constructing and coordinating a particular frame.^28–30^ For example: if a chemistry course has the reputation of “weeding people out,” this *frame* will guide students’ expectations of the course and their actions in it—such as how they engage with assignments, interact with the instructor, etc. Intentionally or unintentionally, an instructor may have framed the course in this way by how they phrased the course description, syllabus, or how they structured the first day of class. Alternatively, an instructor teaching this course may try to change this reputation, taking steps to *reframe* the course to alter students’ assumptions and expectations. In this way, the nature of the frame is dynamic, but the existence of a frame is unavoidable.^28^ Intentional or unintentional, framing directly influences chemistry teaching, learning, and students’ trajectories.^30–34^

The concepts of deficit and anti-deficit framing are particularly relevant for thinking about how frames create inequities in educational outcomes.^35,36^ Despite the extensive evidence about the importance of deficit and anti-deficit framing in education, these concepts are largely ignored in discussions of postsecondary chemistry education and training. To address this, we here perform an integrative,^37^ or interpretive,^38^ systematic literature review, synthesizing the multidisciplinary literature to formulate a comprehensive description of the structure and function of deficit and anti-deficit framing. This theory-building contribution facilitates recognition of the relevance of these perspectives to chemistry education and training. In this way, this work empowers chemists and chemistry educators with knowledge and tools to rationally approach issues of chemistry education and training in ways that simultaneously hold high standards and improve retention, equity, and inclusion.

## Methods

### Research Paradigm

As a chemistry education research study, this work involves disciplinary chemistry knowledge and systematic methods for generating knowledge about chemistry teaching and learning. In particular, this work is informed by a critical realist paradigm^39^ and guided by sociocultural theory^40,41^ and sociopolitical theory.^42,43^ Our analysis is inductively guided by the data sources as well as these theoretical foundations. For example, these theories prompt consideration of the role of systemic and structural factors and how these influence the data analyzed here. These paradigms inform the selection of specific methods, such as reflexivity^44,45^ and consensus discussions.

### Systematic Literature Review

Here, we follow the Preferred Reporting Items for Systematic reviews and Meta-Analyses (PRISMA) guidelines.^46^ PRISMA guidelines ensure methodological transparency in reporting systematic literature reviews.^46^ This process includes: (1) identifying the review topic and defining research questions; (2) identifying possible records from databases; (3) screening records based on inclusion and exclusion criteria; and (4) analyzing the final set of included records.

### Evolution of Project Research Questions

In this work, we initially sought to synthesize and translate the multidisciplinary scholarship around three interrelated ideas: deficit framing, anti-deficit framing, and asset framing. Therefore, the initial research questions that guided the selection of databases and the development of search terms and screening criteria were:

(1) What are the characteristics and attributes of how the education research literature defines deficit, anti-deficit, and asset frames?
(2) In what ways do deficit, anti-deficit, and asset framed teaching approaches differentially impact students from majoritized or marginalized backgrounds?
(3) What attributes of anti-deficit and asset framing can be employed in higher education to promote student success?

However, the inductive orientation of our research approach informed how our project aims developed responsively to features in the data set. For example: the captured records most directly addressed deficit framing, occasionally mentioning asset-based practices in passing as examples of alternatives but not elaborating in detail about how asset-based frames or practices function. Thus, we narrowed our focus to deficit and anti-deficit framing.

### Information Sources, Search Strategies, and Selection Process

In June 2022, we searched five databases using eight search terms (Table 1), yielding 5010 records. Using a systematic review management software platform (Covidence), we removed duplicate records to yield 4289 unique records. Authors J.B., H.H., and K.A. screened the remaining records based on inclusion and exclusion criteria (Table 2). Each record was independently screened by 2 authors, and any disagreements were discussed to consensus. This process yielded 29 records for analysis (see Supporting Information Table S1).

**Table 1:** Databases and Search Terms

Databases				
SCOPUS	Science-Direct	ERIC	JSTOR	Psych-INFO

Search Terms			
“Anti-deficit”	“Deficit-approach” AND education	“Deficit fram*” AND education	“Deficit model*” AND education
			
“Deficit perspective*” AND education	“Deficit thinking” AND education	“Asset fram*” AND education	“Asset-based approach” AND education

**Table 2:** Eligibility Criteria

Inclusion Criteria	Exclusion Criteria
• The record is a peer-reviewed journal article written in English.	• The record is not a peer-reviewed journal article written in English.
• The record’s findings and/or conclusions directly contribute to the understanding of deficit, anti-deficit, or asset-based paradigms, and that is obvious from the title or abstract.	• Upon reading the full record, the findings and/or conclusions do not directly contribute to the understanding of deficit, anti-deficit, or asset-based paradigms.
• Deficit, anti-deficit, and asset-based paradigms are discussed in an educational context, and that is obvious from the title or abstract.	• The record does not contain a definition or description of deficit, anti-deficit, and/or asset-based paradigms.

### Data Analysis

To address our research questions, we used a concept analysis (CA) approach.^38,47^ This involves identifying defining attributes, antecedents (necessary precursors), consequences (resulting outcomes), and additional cases (e.g., model, borderline, related, and contrary cases). Using this framework, J.B., H.H., and N.M.J. analyzed 9 records. Each author independently analyzed each record, identifying features aligned with the concept analysis framework. After analyzing each record, J.B., H.H., and N.M.J. met to discuss interpretations to consensus. Areas of extended discussion often involved differentiating if certain features operated as antecedents or consequences, with many manifesting simultaneously in both categories.

J.B. and H.H. then rotated off this project, and E.B.V. rotated on. E.B.V. independently read the 9 records that had been previously analyzed and reviewed the previously generated features and their descriptions. N.M.J. independently reread these records and discussed them to consensus with E.B.V. In consensus discussions, E.B.V. and N.M.J. noticed that the overlap between antecedents and consequences could be indicative of an inherent cyclical or self-reinforcing structure. E.B.V. and N.M.J. then rereviewed these 9 records and found further evidence to support this. This motivated adaptation of the CA framework and drawing on elements of critical phenomenology^48,49^ and phenomenological variant ecological systems theory^50,51^ to guide our thinking about how systemic and/or structural factors influence individuals’ experiences of framing, and how these experiences inform outcomes. E.B.V. and N.M.J. collaboratively developed a cyclical analytical map representing the general phenomenon and function communicated through the identified defining attributes, antecedents, consequences, and model cases. E.B.V. and N.M.J. then independently read and consensus discussed the remaining records in groups of 1–4 records. This generated additional features and iterative revisions to details of the analytic map but did not substantially alter the overall structure or function it represented. After analysis, N.M.J. rereviewed all records for evidence that disconfirmed the analytic map and found none.

### Quality Considerations

To establish the rigor of these findings, we attend to trustworthiness criteria^44^ for qualitative work, including credibility, transferability, dependability, and confirmability.

#### Confirmability

In all scientific work, researchers personal dispositions influence the nature of the project and its outcomes.^52^ To account for this, we attended to ensuring the accuracy and integrity of our analysis through reflexivity and explicitly establishing credibility and dependability. Our reflexivity—the practice of critical reflection on how our positions, perspectives, biases, and dispositions influenced this work—resulted in practices such as explicitly considering, discussing, and articulating our research paradigm (see: Methods: Research paradigm). Throughout this work, when presented with decisions, we engaged in personal reflection and group discussions, interrogating the evidence and reasoning for our decisions to minimize the influence of implicit or unconscious assumptions and dispositions.

#### Credibility

The credibility of these findings is supported through extensive triangulation, the practice of cross-referencing and establishing consistency between independent sources. We incorporate many levels of triangulation: between researchers’ individual interpretations, between unique analyzed records, and between analyzed records and additional external data sources. For example: we triangulated interpretations between researchers by having multiple researchers independently analyze each data source, followed by detailed consensus discussions. We triangulated findings between analyzed records by iteratively referring back to earlier records throughout the analysis process and by reexamining all data sources for disconfirming evidence after establishing our findings. We also triangulated findings between analyzed records and external data sources, including relevant texts referenced frequently within records (e.g., refs 26 and 53), additional texts that we recognized to be salient to the findings (e.g., refs 54 and 55), and peer-reviewed academic scholarship published after our selection and screening of records (i.e., between July 2022 and June 2025). To make this evidence of credibility transparent to the reader, in our results we provide evidence from multiple sources to support each feature of our interpretations, and in the Supporting Information we include a detailed account illustrating the consistency between these findings and additional external data sources whose content was not used to generate these interpretations.

#### Dependability

The dependability of these findings is supported by the extensive consistency of this general phenomenon across academic research spanning decades and varied local and societal structures. For example: evidence for dependability of these results includes their ability to explain and describe deficit and anti-deficit framing reported in literature grounded in the experiences of K-12 and postsecondary students of color in the US, as well as K-12 students of immigrant families in Belgium, and first-generation postsecondary students in Australia.

#### Transferability

To support the transferability of these findings to diverse contexts, we focus on explicating a comprehensive general overview and explicitly discuss how in situ factors (e.g., social norms, status quo, etc.) influence how these phenomena manifest in specific contexts. In this way, this work provides tools for considering and identifying the relevant contextual features that inform how these phenomena function, such that these ideas can be employed in a variety of contexts where exact details may vary, but the overarching patterns persist. To further support this, in the Supporting Information we include recommended resources that provide more contextually-specific guidance and case studies.

## Results

### (Anti-)­deficit Theory Building in Academic Journal Articles

We identified 4289 unique records (Figure 1A), of which 29 (0.68%) passed all eligibility criteria (Table 2), indicating the record’s findings and/or conclusions directly contributed to the understanding of deficit and/or anti-deficit paradigms in educational contexts. These rates are consistent with typical recommendations for systematic reviews.^38^

This data set includes publications in 25 unique academic journals from 2001 to 2022, spanning six countries (Figure 1B). A complete list of these records can be found in the Supporting Information (Table S1). To illustrate the evidence forming our findings, we include representative quotes from each of the 29 records. The selected quotes come from a variety of sections within the records (e.g., introduction, results, discussion, etc.). While our selected quotes were sometimes informed by other literature, each quote represented an idea generated or synthesized by the record's author(s).

**Figure 1:**
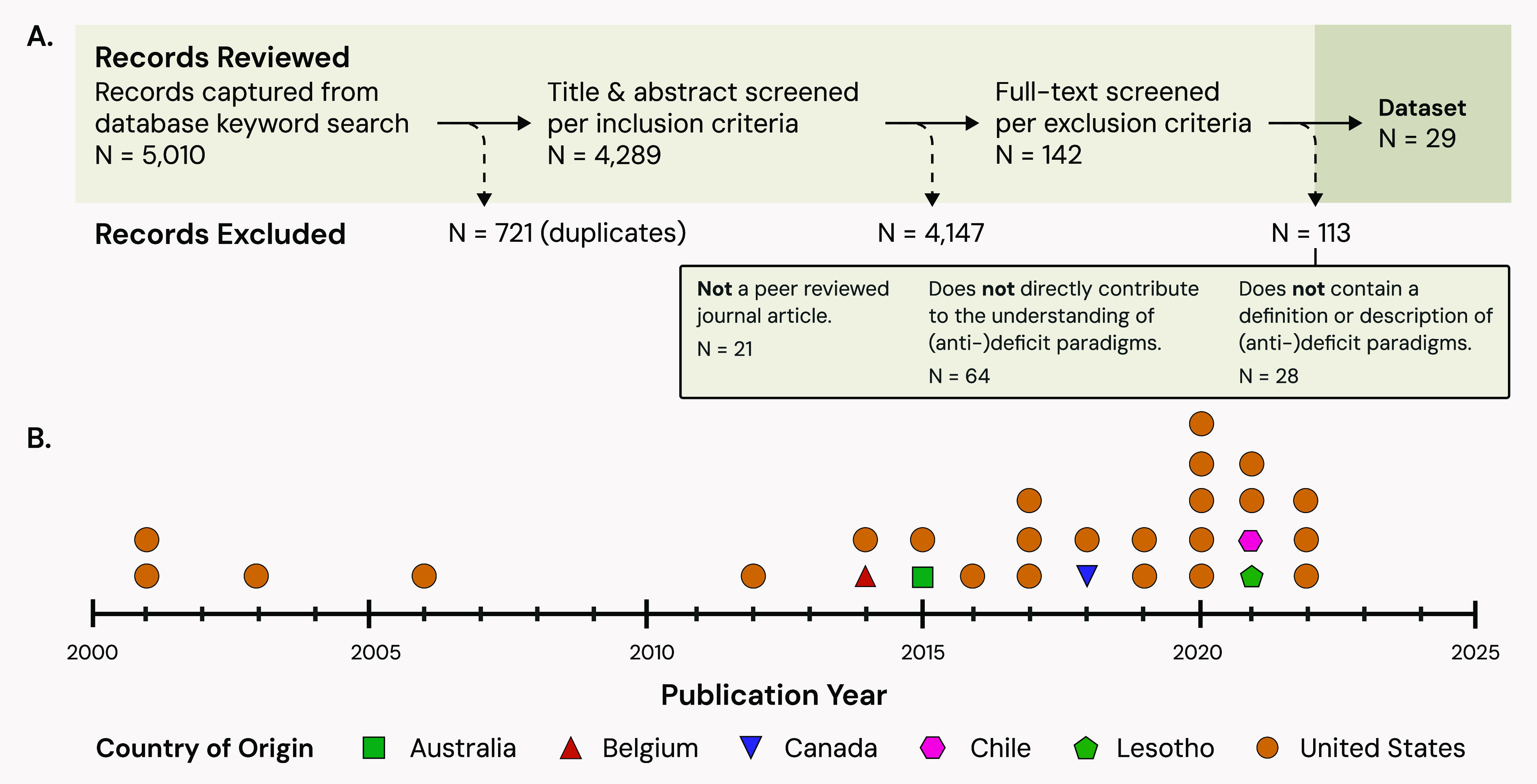
Review process used to identify records (A), and characterization of the resulting data set subject to analysis (B). Note: records captured as Advanced Online Publications (AOP) are reported in panel B based on their AOP year, but in-text references report their final assigned year of publication in the journal issue.

### Structure and Function of (Anti-)­deficit Framing

Stakeholders—such as students, educators, education researchers, administrators, families, communities, and institutions—operate under the influence of local and societal norms, structures, systems, and the cultural and historical contexts that make up their status quo. Because the status quo is socially constructed, stakeholders do also exert influence (albeit to a smaller extent) on the status quo, as indicated by the bidirectional arrows in Figure 2. In other words, the status quo is sociopolitically and socioculturally constructed.

Through stakeholders’ actions, beliefs, and thinking, they construct a frame, which may be deficit- and/or anti-deficit-oriented. All stakeholders are influenced by the frames they operate within. However, this influence manifests differently for stakeholders in different positions and with different degrees of power. As explicated in the following sections, a given frame may incorporate deficit- and anti-deficit-oriented components simultaneously. As such, these are not mutually exclusive categories and are better considered as a spectrum. As model cases, Figure 2 illustrates polar sides of the deficit and anti-deficit framing spectrum.

**Figure 2:**
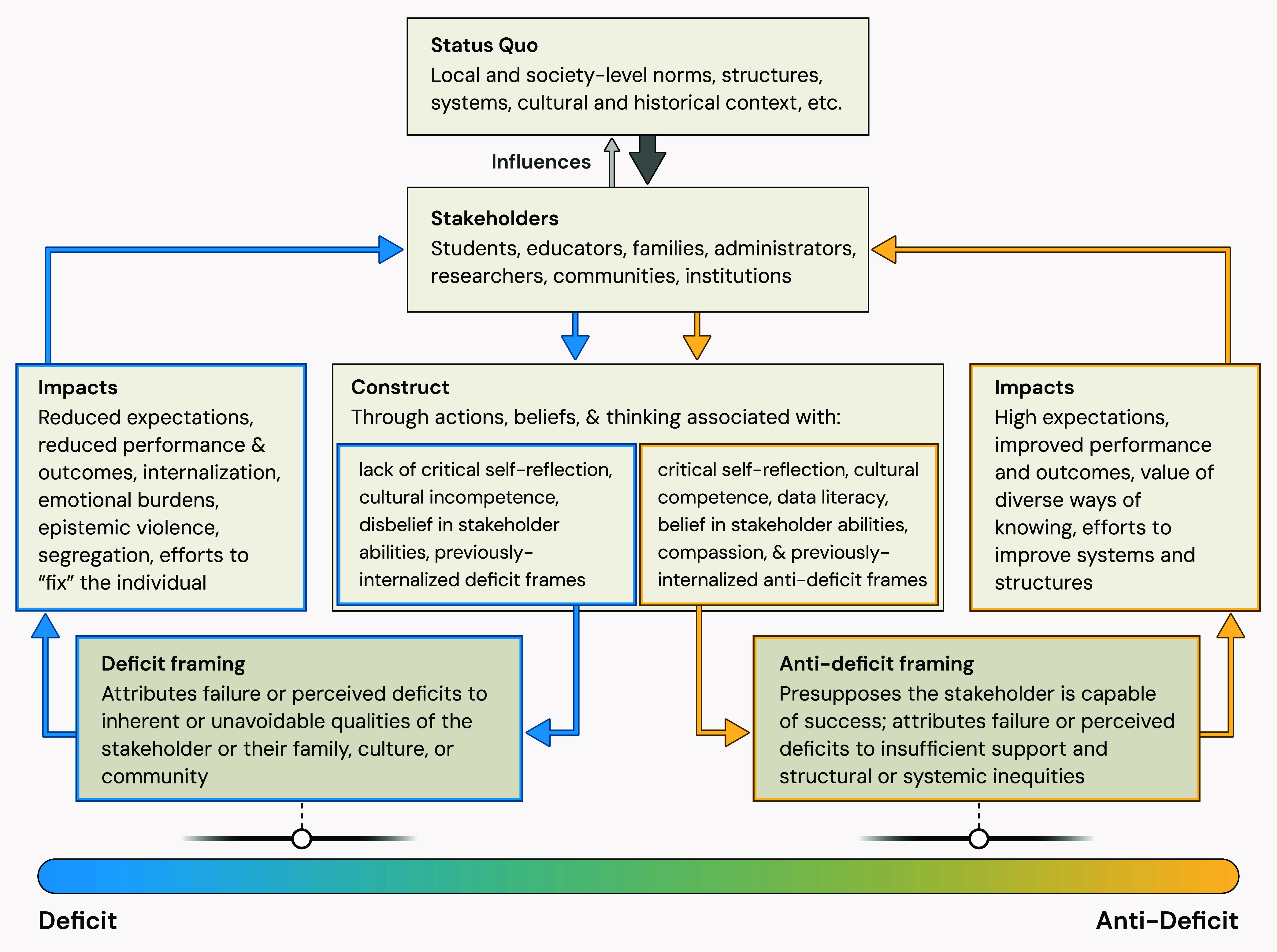
Analytic map of the structure and function of deficit framing (blue) and anti-deficit framing (yellow). Stakeholders are influenced by the status quo but do have agency and (limited) ability to influence the status quo in return. Stakeholders construct a frame, which may be deficit- and/or anti-deficit-oriented, and which impacts other stakeholders in ways that can reinforce the status quo. Frames may incorporate deficit and anti-deficit orientations simultaneously—thus, these orientations are depicted as a spectrum.

### Deficit and Anti-deficit Frames

Fundamentally, deficit frames reflect explicit or implicit assumptions about a stakeholder’s innate or inherent abilities. For example, McKay and Devlin^56^ relay **deficit framed** statements made by academic faculty and staff:

  Low [socio-economic status] barbarians that need to be trained. [...]
  ...you do sometimes hear about how lazy they are, how disengaged they are, how demanding they are. [...]
  ...they cannot hack it and they drop out (ref. 56, p. 5)

In this way, a deficit frame presupposes that failure or perceived deficits result from intrinsic, inherent characteristics of the stakeholder or their background. For example:

  Deficit thinking, regardless of intent, blames individuals, their families, their schools, or their greater communities for the consequences of societal inequities (ref 57, p. 3)
  [...] invoking a deficit perspective [...] infers that students are unsuccessful because of their own intellectual or academic deficiencies, i.e., who they are is not good enough (ref 58, p. 461)
  Deficit thinking is a perspective that attributes students’ underachievement to their failure or deficits as a result of membership in a particular group. [...] Often reported under the guise of "objective research," these explanations have evolved from genetic deficiencies of people to, more recently, inferiority of culture or family values (ref 59, p. 409)

Importantly, a deficit frame holds an individual responsible for deficiencies that are located in social norms, structures, and systems:

  These deficit master-narratives tend to position students as personally and solely responsible for their underachievement (ref 59, p. 404)
  [...] deficit thinking obscures how educators and systemic oppression undermine the success of minoritized students in U.S. schools (ref 60, p. 8)

Alternatively, an **anti-deficit frame** presupposes that the stakeholder is capable of success. For this reason, anti-deficit framing attributes failure or perceived deficits to insufficiencies or inequities in systems and structures:

  An anti-deficit perspective begins with the assumption that students of color are capable of reasoning mathematically and that they bring productive resources for learning mathematics (ref 59, p. 416)
  Anti-deficit perspectives locate the source of academic problems within institutional structures that work to limit access to educational opportunities. The focus is on the assets that students bring to the classroom, rather than what they lack (ref 61, p. 946)

Despite their opposing orientations, deficit and anti-deficit frames are **neither binary, nor static.** A stakeholder may employ components of both frames simultaneously or intermittently:

  [...] in practice, teachers are more likely to blend these [perspectives] than to enact a purely deficit or anti-deficit approach (ref 62, p. 98)
  Although he described diversity as an “asset” in the classroom, he also admitted the pressure teachers feel to ignore diversity and focus on test-taking skills. Moreover, he suggested that some cultures value education less [...] Thus, even a teacher with a strong commitment to diversity fell victim to deficit thinking in some respects (ref 63, p. 15)
  [...] it was difficult for most educators to reconcile their deficit perspectives with their desires to help kids. For example, immediately after a discussion of the need to “salvage” students, [...] one administrator at Kendrick’s school asked, "Are we offering them what we need to offer them? Do we need to change this? Do we need to change that?" (ref 64, p. 19)

In this way, the frame a stakeholder employs may conflict with their intentions. For example:

  Another way in which my approach placed a deficit orientation to the students’ work is how it expected the students use formal language [...] which went against my intended goal with the task of providing students with an alternative way to show their understanding (ref 65, p. 10)
  [...] "deficit-creep" refers to the subtle infiltration of deficit perspectives that can show up in the creation and/or implementation stages of equity and achievement gap policies. [...] it operates in subtle ways among practitioners despite having the language, knowledge, and goals of doing social justice work (ref 66, p. 9)

### Deficit Framing: Impacts on Stakeholders

As summarized in Figure 3, deficit frames have a variety of impacts on stakeholders.

One illustrative example is how deficit framing can result in **reduced expectations**. For example:

  The teaching style that is used when teaching working-class students in working-class schools often involves rote behavior [...] [that] is rooted in the deficit assumptions that working-class children are not able to develop critical skills (ref 67, p. 146)
  [...] the [prospective teachers] described how their mentor teachers often expressed deficit narratives and attached dehumanizing labels to students in the classrooms where they had been placed. Deficit perspectives led to student blaming and lowering of expectations (ref 68, p. 4)
  [...] she and other administrators felt "noble" about working in high-poverty schools because they kept the children "warm, safe, and on a regular schedule" and that they thought the poor academic performance of the students was "inevitable and not anyone’s fault" (ref 69, p. 252)

In positioning some stakeholders as inherently incapable of success, a deficit frame also lowers expectations of other stakeholders. For example, presuming particular students are inherently incapable of success **absolves responsibility** but also **denies agency** of educators and educational institutions:

  Part of the appeal of deficit models lies in shifting responsibility away from oneself. No one wants to be implicated in a system that maintains major barriers to academic and personal achievement (ref 70, p. 149)
  Deficit thinking stems from the ethnocentric notion that the beliefs and standards of the dominant group are inherently correct. [...] In this way, schools construe cultural, social, and linguistic differences as problems that originate in the home and over which educators have little influence (ref 71, p. 71)
  [...] it presupposes that an individual pupil is (strongly) determined by his or her home environment. From that perspective pupils’, parents’, and even teachers’ agency only seems to play a minor role (ref 72, p. 811)

These deficit beliefs and thinking can implicitly prime assumptions about individuals from groups believed or thought to be deficient, resulting in **overdisciplining** and **overlooking** the abilities of individuals from those groups:

  Deficit discourses may give rise to *deficit noticing*, wherein teachers attend almost obsessively to the errors and shortcomings of students of color; interpret errors and shortcomings as evidence of deficiencies in students, their families, or their cultures (ref 62, p. 95)
  [...] inner-city students often are seen to be troublemakers even prior to creating any disruption in class; acknowledging this then, what happens when there is a disagreement the student has with an educator, would it be tainted with prejudgement? (ref 67, p. 147)
  [...] if teachers assume Black children come to school with a series of individual and community "deficits" they may react more strongly with disciplinary behavior toward those youth, whether necessary or not (ref 73, p. 2)

Correspondingly, deficit framing can result in **reduced performance or outcomes**:

  [...] marginalized students are limited and constrained due to the low standards and expectations set for them by well-intentioned educators; who unconsciously hold deficit perspectives (ref 67, p. 137)
  Deficit thinking inhibits educators from adopting practices that increase minoritized students’ achievement in the K-16 educational pipeline (ref 60, p. 8)
  The result of this pervasive deficit approach is that students from low-income homes and students of color routinely and overwhelmingly are tracked into low-level classes, identified for special education, segregated based on their home languages, subjected to more and harsher disciplinary actions, pushed out of the system and labeled "dropouts" (ref 69, p. 236)

Critically, individuals often **internalize** the deficit frames placed upon them. For example:

  At first I was like, we must be dumb, we are not smart like the other kids. Latinos are different. So I started to act dumb and not do my work and stuff like that. What does it matter, right? Like, if you are not smart, [you are] not going nowhere (ref 74, p. 68)
  Though students occasionally expressed reasons for this anger that did not reflect self-blame, they often adopted educators’ deficit perspectives of themselves (ref 64, p. 14)
  Even educators who themselves are from culturally, linguistically and economically diverse backgrounds often hold deficit beliefs because they have assimilated and adopted mainstream perspectives (ref 71, p. 70)

In effect, the deficit frame focuses attention to **fix the individual** such that they conform to existing systems and structures rather than to identify and address any underlying issues in those systems and structures. For example:

  These deficit master-narratives tend to position students as personally and solely responsible for their underachievement. This positioning then leads to "solutions" that focus solely on "fixing" the students (e.g., teaching "grit" to Black students and students in poverty) while leaving the unjust system intact (ref 59, p. 404)
  At the institutional level, deficit thinking implies that students must address their own underpreparedness through initiatives outside the curriculum to "level" their knowledge (ref 75, p. 2)
  [...] consistent with deficit thinking, grit and growth mind-set focus on shifting people’s perspective toward challenges they face and instilling the belief that individuals can "grow" desirable characteristics or attitudes. [...] encouraging people to adapt to broken systems instead of questioning them (ref 70, p. 138)

This focus on fixing the individual often corresponds to **pressure to assimilate** to dominant societal norms and primarily recognizes the knowledge assets that align with the mainstream status quo. For example:

  ‘Difference’ is all-too often associated with deficit, dysfunction, and disadvantage (ref 56, p. 3)
  [...] deficit thinking operates under the assumption that a given population exists in a state of need. Most often, the ideal means of addressing this need is for the people with the perceived deficit to apply themselves, to conform, or otherwise to assimilate to the dominant culture (ref 70, p. 139)
  Our results help us to reflect on the production of subjectivities that these [academic] programs entail, which permeates the discourse of the participants, who are expected to reproduce or conform to a set of "desirable" entry-level traits (ref 75, p. 9)

This constitutes a form of **epistemic violence** and **cultural violence**, which denies or suppresses some forms of knowledge and aligns specific cultural standards with success:

  [...] the education received at home (e.g., with respect to language, values, and norms) had to be ‘subtracted’ so that the school could add the ‘right’ language proficiency, attitudes, skills, and so on (ref 72, p. 798)
  Deficit master-narratives include assumptions about the kinds of knowledge considered to be productive, the way learning happens, and the kinds of students who can be successful (ref 59, p. 407)
  These [Black] students [...] come to associate or equate academic achievement with "acting White" [...] Thus, gifted African American students may underachieve deliberately, refuse to be assessed for gifted education services, and refuse placement in gifted programs (ref 76, pp. 55–56)
  Altogether, tests privilege a very narrow slice of mathematical competence while ignoring vast amounts. The erasure of expertise may be more profound for students from oppressed communities, who often have rich histories of community-based mathematical practices that look very different from pencil-and-paper tests (ref 61, p. 949)

In this way, deficit framing often causes severe **psychological and emotional harm** to stakeholders:

  It hurt me real bad at first ... thinking that Latinos and myself are dumb or less than them. It still kind of hurts (ref 74, p. 68)
  [...] teachers' ideas about the low teachability of ethnic minority pupils are reflected in pupil’s feelings of futility, demotivation, and even psychological disengagement from education (ref 72, p. 812)
  Perhaps the worst consequence of deficit thinking among educators is the impact it has on the social-emotional and psychological development of Black students (ref 76, p. 55)

### Anti-deficit Framing: Impacts on Stakeholders

As summarized in Figure 3, anti-deficit framing can result in stakeholders holding and/or meeting **high expectations**. For example:

  Once they saw that all students could achieve at much higher levels than previously accepted in their districts as the (deficit-driven) norm, they all began to articulate higher expectations for their students’ success (ref 69, p. 254)
  At the core of these frameworks is the belief in high expectations for all students, even when taking into consideration that achievement is different for everyone (ref 77, p. 50)
  One [prospective teacher] told us about the high expectations that her mentor teacher held for her students. When the mentor teacher’s students struggled in mathematics, she refused to attach labels to them (ref 68, p. 4)

**Figure 3:**
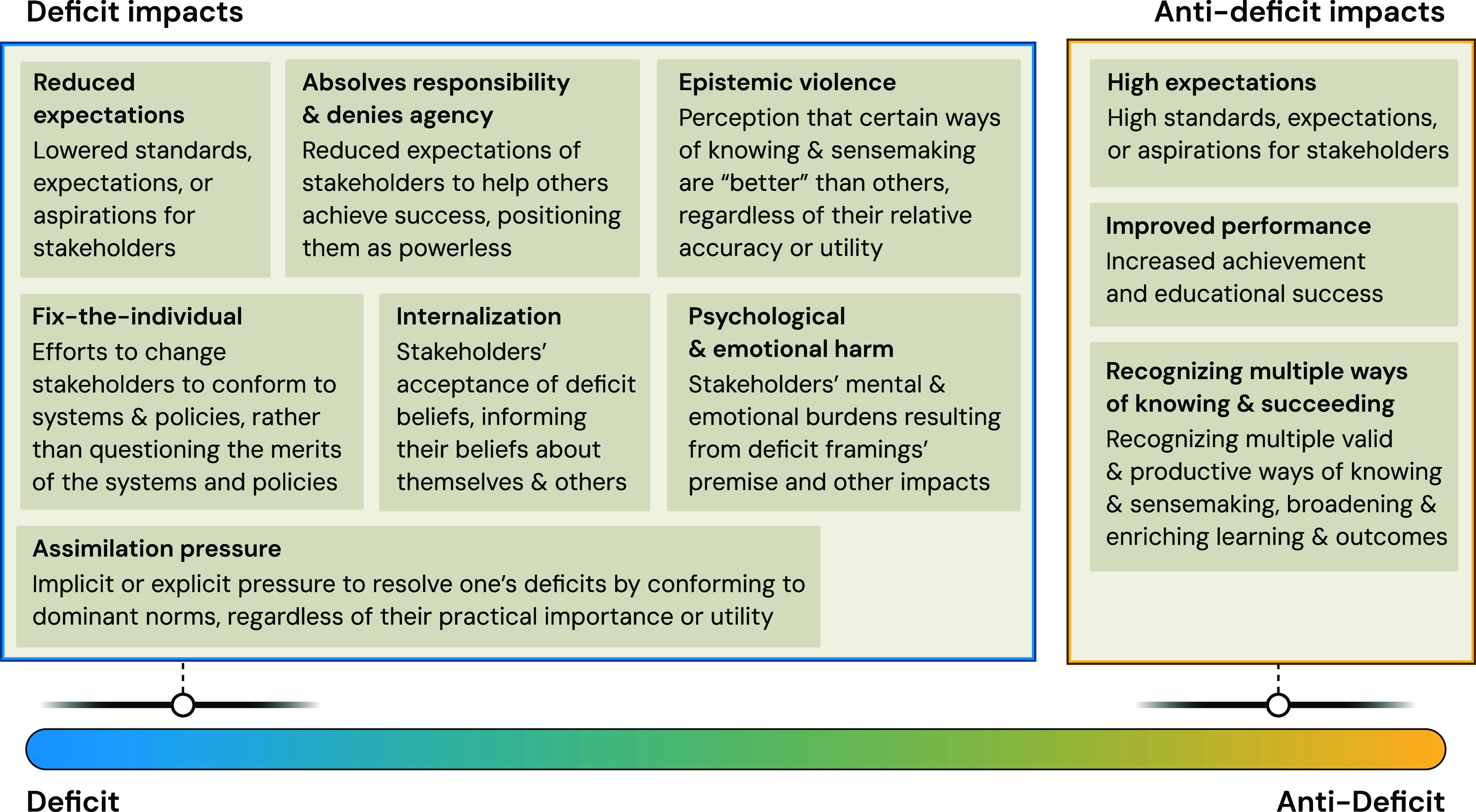
Mechanisms of how deficit and anti-deficit frames and framing impact stakeholders.

Correspondingly, anti-deficit framing can result in **improved performance or outcomes**. For example:

  Preliminary research in some of these districts points to the possibility that the superintendents have found ways to resist deficit thinking and, thus, to make strong, demonstrable progress toward educational equity (ref 69, p. 238)
  Faculty who reinforce the view that all students bring unique talents and perspectives to engineering classrooms not only positively impact first-generation college students, they also positively influence access to resources for all students (ref 78, p. 19)
  [...] when students are given the right kinds of support, many students who would otherwise be assigned to remedial courses can be successful in credit bearing courses—they can "jump the sequence" (ref 61, p. 947)

Importantly, these high expectations and improved outcomes often **recognize multiple ways of knowing and succeeding** beyond those reflected in the dominant or mainstream status quo. For example:

  [...] framing learning as interactive supports teachers to interpret and build on students’ diverse ways of being as resources not only for individuals’ learning but also for collective advancement, thereby disrupting hierarchical, deficit discourses that harm all students, especially students of color (ref 62, p. 104)
  [...] anti-deficit perspectives recognize the productivity of the knowledge that students of color gain from their experiences in and out of the classroom (ref 59, p. 416)
  Students’ mathematical ideas can be incorporated in instruction as the teacher makes a point to include and build on students’ ideas to help students make meaning of concepts and experience mathematical success (ref 68, p. 5)

### Self-reinforcing Cycle

The impacts of deficit frames often interact to reinforce and amplify those same frames, perpetuating and intensifying the deficit framing cycle:

  A deficit perspective produces a deficit interpretation and a deficit story about Adriana’s sense making that feeds back into existing deficit narratives about the mathematics ability of students of color (ref 59, p. 425)
  [...] the cycle of deficit pedagogy can lead to under-achievement, which reinforces the stereotypes about the capabilities of racially marginalized students (ref 67, p. 150)
  Because many educators also lack sufficient cultural knowledge to understand intercultural dynamics in classroom settings and typically do not have access to professional development that helps them develop this knowledge, deficit beliefs are often reinforced through professional practice (ref 71, p. 72)
  [...] educators describe deficits, deficiencies, limitations, [...] explain these deficits by locating them in such factors as limited intelligence of dysfunctional families; [...] predict the perpetuation and accumulation of the deficits; and, finally, [...] prescribe educational interventions designed to remediate the deficits. This cycle has become self-perpetuating as the system in place in traditional U.S. schools, by design, produces failure for some students (ref 69, p. 236)

There is also a self-reinforcing mechanism inherent in anti-deficit framing. For example:

  [...] as they experienced academic success with students for whom they did not previously believe this was possible, as they experienced incremental success with these latter students, they year-by-year pushed expectations and goals higher and higher (ref 69, p. 257)

However, the momentum incumbent in existing deficit frames suggests the self-reinforcing nature of deficit frames is “stronger” than those of anti-deficit frames, reflected in a graphical representation presented and described by Louie et al.:

  To engage in alternative frames therefore takes substantial and ongoing work, including work at the level of individual teachers *and* work at the level of systems and institutions. This is reflected [...] with a large arrow indicating the strong influence of culturally dominant frames [...] and a smaller arrow reflecting a weaker but existing influence in the opposite direction—and possibilities for resistance (ref 62, p. 98)

### Societally-imposed Deficit Frames

Deficit and anti-deficit frames are not equally prevalent. Each stakeholder is unavoidably embedded in a larger historical, cultural, and societal context. The norms and status quo of the stakeholder’s context thus prime the frames they employ.

In other words: a stakeholder is never a “blank slate,” their societal position “bakes in” deficit frames that inevitably influence the stakeholder. Figure 4 summarizes key features of the societally-imposed status quo, which preferentially prompt deficit frames. For example:

  [...] all too often the structures and normative practices of our institutions seem to facilitate deficit perspectives of students (ref 61, p. 953)
  Some educators who (un)­consciously hold deficit assumptions about students, are players in a game that they may not be aware of yet but consistently participate in (ref 67, p. 139)
  Because of the insidiously pervasive deficit thinking in which superintendents, along with the vast majority of other educators including teachers and principles, have been more or less marinated throughout their careers, these superintendents tend to view the broad-scale underperformance of children of color and children from low-income homes in their schools as inevitable, something that is not within their power to change (ref 69, p. 237)

**Figure 4:**
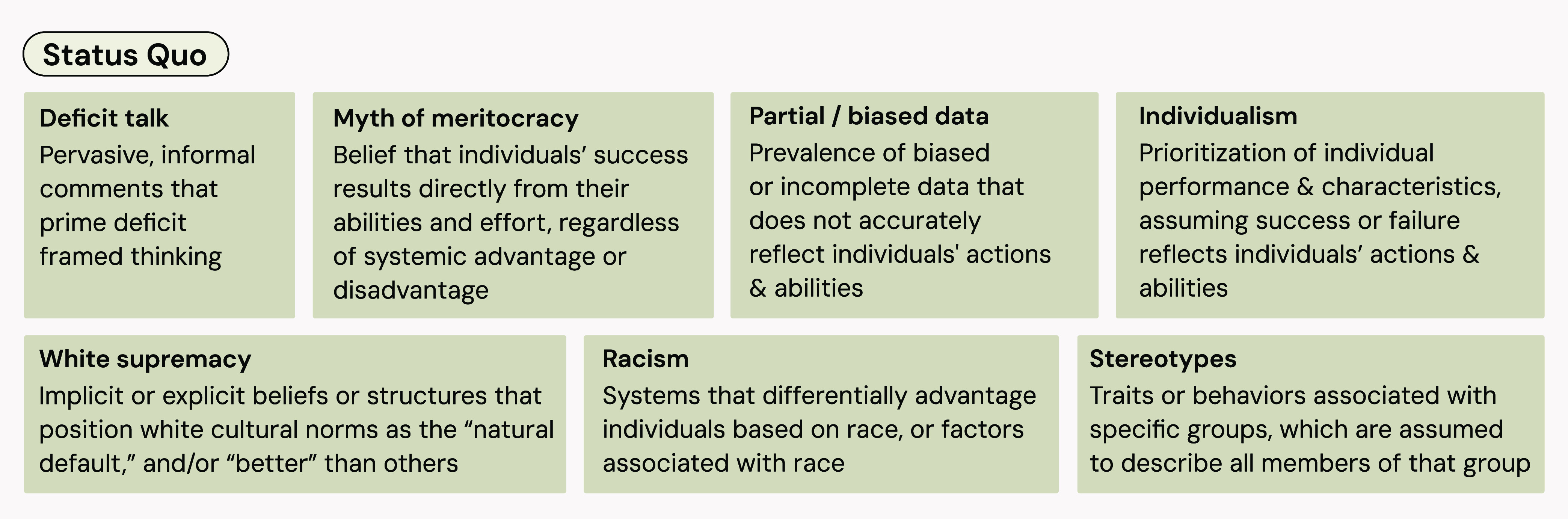
Features of the societally-imposed status quo that prompt deficit frames.

In this way, individual stakeholders are not “at fault” for the existence of societally-imposed deficit frames or the influence these frames have on stakeholders. For example:

  [...] neither institutions nor students are in deficit; rather, there is an existing sociocultural incongruity between middle-class higher education institutions and students from low [socio-economic] backgrounds that needs to be bridged (ref 56, p. 4)
  [...] rather than locate deficit views primarily within biased individuals, we focus here on deficit discourses as socially, culturally, and historically produced (ref 62, p. 96)

However, these deficit frames are constructed, perpetuated, and sustained collectively through stakeholders’ actions, thinking, and beliefs, which in turn inform how stakeholders construct and support existing systems and structures. For individual stakeholders such as chemistry educators and students, deficit framed beliefs and thinking can manifest through actions described previously (see: Deficit Framing: Impacts on Stakeholders), as well as through actions such as **deficit talk** and **deficit noticing**:

  Through everyday storied teacher talk, I was "taught" that the reasons for racial disparities in student achievement boiled down to poor parenting and/or cultural deficiencies (ref 79, p. 95)
  [...] I realized that my approach placed a deficit orientation to the students’ work [...] the rubric was set up to take off points for lack of clarity or inconsistencies. In some ways this shifted my focus onto students’ shortcomings (ref 65, p. 10)

For organization-level stakeholders (e.g., communities, institutions), actions, thinking, and beliefs can manifest through structures, such as those that legitimize inequitable policies or (over)­rely on **partial or biased data**:

  Limiting measures of educational success, also conceptualized as achievement, to performance on exams or overall college GPA, often leaves out consideration of other potential data sources. This narrow perspective tends to perpetuate the systems of power and privilege that are already in place (ref 57, p. 4)
  A Mangrove teacher described the assessment system as unfair [...] A teacher at Southside Elementary, which has a sizable population of Haitian students, also saw the test as culturally biased [...] In general, however, teachers were focused on classroom strategies to improve students’ achievement rather than questioning the fairness of the tests (ref 63, pp. 15–16)
  Educators [...] reiterating the importance of having data to support placement decisions [...] though such documentation tended to be subjective in nature and lacking validity and reliability. District-level administrators accepted these data as evidence of students’ deficits and justification for their removal without scrutinizing (ref 64, p. 17)

The exact nature of the dominant social norms and status quo informs which stakeholder attributes are prone to deficit framing (i.e., in need of “fixing”). Internationally prevalent and persistent features of the status quo include **individualism** and the **myth of meritocracy**:

  This [deficit thinking] rationale, which overstates individual aptitude, tends to guide institutional support initiatives toward bridging general competencies and subject matter contents, but it overlooks the role of structural inequalities (ref 75, p. 9)
  Perpetuating individualized notions of student success reinforces meritocratic ideologies that [...] dismiss the role of racism, sexism, and other forms of oppression that undermine the success of Black female undergraduates and other minoritized college students (ref 60, pp. 15–16)
  [...] social and ethnic inequalities in educational outcomes are rationalized and legitimated through the liberal rhetoric of a meritocratic school system, in which individual merit is coined as the main determinant for educational success (ref 72, p. 798)

These features stem from and reinforce a status quo where White culture and characteristics are viewed as “normal,” neutral, or naturally better than others—a latent manifestation of **White supremacy**. For example:

  Latino students are often positioned as "culturally different," which suggests that the dominant culture is "normal" and that students who come from different cultural communities are deviating from the norm (ref 74, p. 70)
  [...] the deficit perspective holds white, middle-class norms as the ideal and measures all students against this standard (ref 63, p. 16)
  This perspective prompts the interrogation of seemingly neutral discourses with racialized impacts, for example, discourses that treat particular student behaviors as normal and acceptable (i.e., behaviors associated with White, middle-class norms) and other kinds of behavior as deviant, inferior, or wrong (i.e., all other behaviors) (ref 62, p. 96)
  Colby highlighted [...]: When it came time for me to formulate a definition of student success, I struggled ... because I later realized that a lot of what I understood about student success was based on White, middle-class culture and dominant ideologies of education and success (ref 60, p. 18)

As a result, deficit framing narratives often reflect and perpetuate **racism** and racial and ethnic **stereotypes** of groups whose race and ethnicity do not align with the status quo:

  This deficit perspective regarding cultural diversity keeps educators from recognizing the gifts and talents of African American students (ref 76, p. 52)
  [...] a teacher who organizes her work around "closing the racial achievement gap" implicitly frames Black, Hispanic, and indigenous students as mathematically lacking and White students’ achievement as the standard by which they should be measured [...] This framing makes her more likely to attend closely to Black, Hispanic, and indigenous students’ errors without attending to their knowledge or strengths (ref 62, p. 97)
  A striking finding is that the (most) stigmatized minorities—Turkish and Moroccan families—to some extent internalize and perpetuate these social representations [...] The following pupil talks about her parents idea to look for a school with fewer pupils that have the same Turkish background as herself. 'That’s what she [mother] said: "if there are a lot of Turkish girls in your class, I’ll just change you to another class. The less Turks, the better"' (ref 72, p. 810)

Analogous patterns operate along other dimensions of dominant norms and status quo, such as **socioeconomic status** or other locally relevant characteristics:

  [...] the view that other socioeconomically-marked varieties of English reflect inherent deficiencies [...] remains a widely accepted belief (ref 80, p. 31)
  When first-generation college [...] students for whom neither parent who has earned a four-year college degree–are studied, researchers often explicitly or implicitly adopt a deficit mentality (ref 78, p. 2)
  The principal at Langston Elementary referred to students from a low-income Mexican barrio as "the Atwood kids," for example, and Park Elementary staff referred to low-income students from outside the immediate, wealthy neighborhood as "the apartment kids." Other participants identified these terms as carrying a derogatory connotation, without specifying a particular racial/ethnic or socioeconomic group (ref 63, pp. 9–10)

### Anti-deficit Framing: Disrupting Deficit Frames

Given the ubiquity of societally-imposed deficit frames and their self-reinforcing momentum, employing anti-deficit frames requires challenging or disrupting existing societally-imposed deficit frames. Developing the tools to do so requires opportunities to engage in **critical reflection** and develop **critical consciousness**:

  The success schools achieve at recruiting and retaining diverse students in gifted education depends heavily on critical self-examination (ref 81, p. 224)
  What we can all do, however, is acknowledge deficit explanations and examine them critically. Invariably, this illuminates possibilities that have eluded us, including strategies that focus on student strengths (ref 82, p. 43)
  [...] we need to recognize deficit thinking when we see it. This can be difficult because such ideas are all around us and we are accustomed to them. It requires critically reflecting on how we are socialized into perpetuating these myths (ref 70, p. 155)
  [...] teachers need to engage in critical listening to, and reflection on, informal teacher talk about students as a means of surfacing underlying beliefs and biases about racial/cultural "others" ... and for developing a critical consciousness of the ways in which informal deficit-based teacher discourse can act in unseen ways to rationalize and perpetuate existing educational disparities (ref 79, p. 97)

Critical reflection and critical consciousness enable stakeholders to **recognize stakeholder assets**, affirm their **belief in stakeholders**’ **abilities**, and engage in **compassion** and **empathy**:

  [employing an anti-deficit framework, Oscar] treated these student attributes not as problems to be managed but as resources to be leveraged. Moreover, Oscar was able to interpret very different qualities as strengths; there was no one way of being or thinking to which he expected every student to conform (ref 62, p. 101)
  Such an approach is inclusive, and focuses on children’s home language and interests as assets rather than deficits [...] [and] seeks to include rather than exclude diverse language backgrounds in classroom settings (ref 80, p. 34)
  What happened [...] stresses the importance of diversifying my approaches to assessment. Moreover, I now see that in addition to "believing" that students have great ideas, an anti-deficit perspective asks that I give students the opportunity to articulate those ideas, so students can recognize that they do indeed understand the material on some level. The next step is to support students in building on those ideas to strengthen their understanding (ref 65, p. 11)

This is closely connected to the stakeholders’ **(multi)­cultural competence**:

  [...] educators are most responsive to diverse students when they are competent or striving to become competent in the students’ culture. Just as teacher incompetence in a subject area hurts students so, too, does multicultural incompetence (ref 81, p. 221)
  It is key to understand that differences—especially differences from us and how we learn, speak, or listen—are not deficits. [...] Learners’ experiences, interests, and lives shape their perspectives [...] and these points of view must be made an integral part of teaching (ref 70, p. 155)

and involves **data literacy** skills, such as the ability to accurately identify inequities in data, policies, and systems:

  [...] schools cannot hide behind test scores; instead, they must also examine how policies and procedures disparately impact the recruitment of minority students. [...] These policies must be evaluated, changed and, if necessary, eliminated (ref 81, p. 220–221)
  Disaggregating data is necessary, as is analyzing those data with a just framework that dismantles racial hierarchies and carefully considers the sources of data used to understand those inequities. The frameworks we choose affect our analysis; we must avoid the common trap of assuming that quantitative data and data analysis are free from bias (ref 57, p. 8)

Developing these capacities requires **high-quality training** and **structural support**:

  [...] the school district should provide opportunities for continuing professional development in gifted and multicultural education. More specifically, faculty members and other school personnel must be encouraged and given opportunities by administrators to participate in workshops, conferences, university courses, and so forth (ref 81, p. 224)
  Developing this level of cultural competence requires a transformative journey that takes educators beyond cultural awareness and knowledge to a place where deficit beliefs and practices can be explored, challenged, and changed (ref 71, p. 90)
  An interesting path to explore are training programs that invest in intercultural competencies preparing and strengthening teachers to teach in ethnically, religiously, and culturally diverse classrooms. This could help teachers understand what is happening in their classroom and how to effectively teach in a 'multicultural' classroom (ref 72, p. 813)

Importantly, the pre-existing embeddedness of deficit framing in the mainstream status quo, coupled with the self-reinforcing nature of framing cycles, means that **ongoing reflective vigilance** is needed to sustain anti-deficit framing:

  [...] the work of anti-deficit reframing must be ongoing for everyone [...] because dominant deficit discourses have a tenacious hold on our field and will continue to influence all of us (ref 62, p. 105)
  [...] it may be useful to think about the reduction of cultural deficit beliefs as a life-long project. [...] Interventions in the form of continuing teaching support groups [...] catching inequitable action that follows from habit, inattention, or unconscious biases will require vigilance (ref 73, pp. 9–10)
  The only way to dismantle deficit thinking, an institutionalized worldview that is woven into our society, our colleges and universities, and our teaching, is piece by piece (ref 70, p. 155)

### Reframing vs Frame Shifting

Importantly, the anti-deficit frame fundamentally asserts that stakeholders are capable of achieving high expectations and attributes any lack of success to societal histories, systems, and structures. In this way, it also requires careful attention to reframe without simply “re-directing” the existing frame. For example:

  [...] even when the training is aimed to arrest deficit thinking about students, [often it] is based on the teacher deficits variant. Like remedial programs for students, professional development programs "fix" teachers by identifying what they don’t know or do and telling them how to do it (ref 82, p. 45)
  These objectives tend to position students and teachers as the sites in need of intervention, and in doing so they employ deficit frameworks [...] This impacts all aspects of policy implementation including the trickle down of deficit perspectives from policy makers to school leaders to teachers, and so on (ref 66, p. 9)

Arguably, shifting the direction of a deficit frame still accepts the deficit framing premise: that individuals are “at fault,” rather than societal systems and structures. In this way, “frame shifting” changes which stakeholders are expected to compensate for the deficiencies of societal systems and structures without addressing the fundamental deficiencies themselves. In other words: it “shifts the blame” while leaving intact the underlying systemic issues that created a reason to place blame. In contrast, “reframing” involves a more substantial re-envisioning of the problem and of potential solutions:

  Developing and sustaining anti-deficit noticing requires changes not only to individual teachers as isolated actors, but also changes to the systems that make deficit framing seem normal or necessary (ref 62, p. 105)
  Deficit models view students as perpetual lacking and at fault. This belief is neither healthy nor accurate. Instead, we need to remain open to broader ways of engaging students and of thinking about their lives, consider what power they really have to effect change, and where we share some responsibility (ref 70, p. 150)

## Discussion

Here, we have illustrated how the deficit framing cycle operates automatically when stakeholders’ identities or behaviors deviate from the status quo. Given the significant historical and current role of racism in the sociocultural and sociopolitical construction of the status quo (Figure 4), it is no surprise that racism is closely intertwined with the very foundations of deficit framing. The development of deficit and anti-deficit framing as concepts is heavily grounded in scholarship surrounding the educational experiences of people of color, particularly Black youth in the United States.^64,76,81^ The unique racial history of the US informs the societal norms, systems, and overall status quo that influence the ways in which their lives and educational experiences are affected by deficit framing. Community-level variation in racial histories and cultural norms results in varied manifestations of deficit framing. However, globally, there are widely conserved status quo trends, such as those reported in records examining the experiences of low socioeconomic status undergraduate students in Chile^75^ and Moroccan and Turkish immigrants in Belgium.^72^ The concept of deficit framing is deeply tied to the sociopolitical and sociocultural construction of race, the racialized nature of the status quo, and the uniquely strong impact that deficit framing has on stakeholders from oppressed racial groups. However, these ideas also apply to other dimensions of identity and behavior. Here, we aim not to appropriate these ideas but to recognize their origins and existence as tools for making education more accepting and inclusive for all individuals and especially people of color. Toward this, here we discuss thematic implications of these findings and their relevance to postsecondary chemistry education contexts.

### It Is Impossible To Be “a-Deficit”

Intentionally or unintentionally, all situations and activities are framed in some way.^28^ The strong influence of the status quo in the systems, structures, and actions of stakeholders creates an “automatic” deficit frame for some stakeholders, regardless of the intention. As a result, it is impossible for a stakeholder to not engage in some degree of either deficit or anti-deficit framing. In other words: it is impossible to be neutral or “a-deficit.” Given the implicit and automatic nature of deficit framing, constructing alternative frames requires an active, intentional, and sustained effort. Without this, frames will often “default back” to deficit orientations.

This underscores the importance of recognizing that “deficit” and “anti-deficit” are attributes of the frame a stakeholder constructs, not attributes of the stakeholder. In other words: one is not a “deficit or anti-deficit person.” Rather: through one’s thinking, actions, and behaviors, one constructs deficit- and anti-deficit-oriented frames that can change fluidly over time. Even individuals who consciously strive toward anti-deficit-oriented approaches can experience “deficit creep,”^66^ where their thoughts or actions unintentionally revert back to societally imposed deficit frames.

One tool for constructing and sustaining anti-deficit-oriented frames is through the thoughtful selection of frameworks, theories, and/or pedagogies that structurally “build in” an anti-deficit orientation. Because “how a problem is defined determines how it is approached,”^70^ the guiding orientation of frameworks, theories, and pedagogies tangibly influences the resulting frames and actions.

For example, Grit^83^ and Growth Mindset^84^ are frameworks commonly used by well-meaning chemistry educators aiming to improve student experiences and course outcomes. This is particularly important in General and Organic chemistry courses, which often act as gatekeepers limiting students’ ability to pursue chemistry and STEM.^85–87^ However, Grit and Growth Mindset frameworks can function to “put the blame for a lack of learning on students, ignoring the systemic issues that impact them”.^70^ In this way, using Grit or Growth Mindset alone as frameworks to inform one’s teaching may result in the perpetuation of deficit frames, where students who are already “victims” of the system are required to do more or work harder than others, in order to compensate for systemic issues. This would suggest a need for careful, critical reflection and working to ensure structural supports are also in place for chemistry students to address the root causes of systemic inequity.

Similarly, using Grit or Growth Mindset to inform the design of a chemistry education research project may focus researchers’ attention on developing “best practices” that fundamentally work to promote learners’ assimilation to dominant norms. To support an anti-deficit approach and outcome, a chemistry education researcher might intentionally select an anti-deficit oriented framework. Or, if the project merits using a framework such as Grit and/or Growth Mindset, a chemistry education researcher might develop a conceptual framework^88^ to integrate an additional theory that structurally provides an anti-deficit orientation.

Many of the records analyzed here refer to specific frameworks, theories, and pedagogies that facilitate or “prime” anti-deficit-oriented frames, as well as some that implicitly or inherently prime deficit-oriented frames. Examples of these are shown in Figure 5, and a detailed list is included in SI Table S2. Notably, many records refer interchangeably to “asset framing” and “anti-deficit framing” or refer to “asset-based approaches” as ways to structurally incorporate an anti-deficit frame. In this way, asset and anti-deficit framing are closely related ideas. We have chosen to primarily use the term “anti-deficit,” as it more directly reflects the need to actively work to challenge entrenched, pre-existing frames with deficit orientations. Simultaneously, we highlight asset-based approaches as useful tools for doing so.

**Figure 5:**
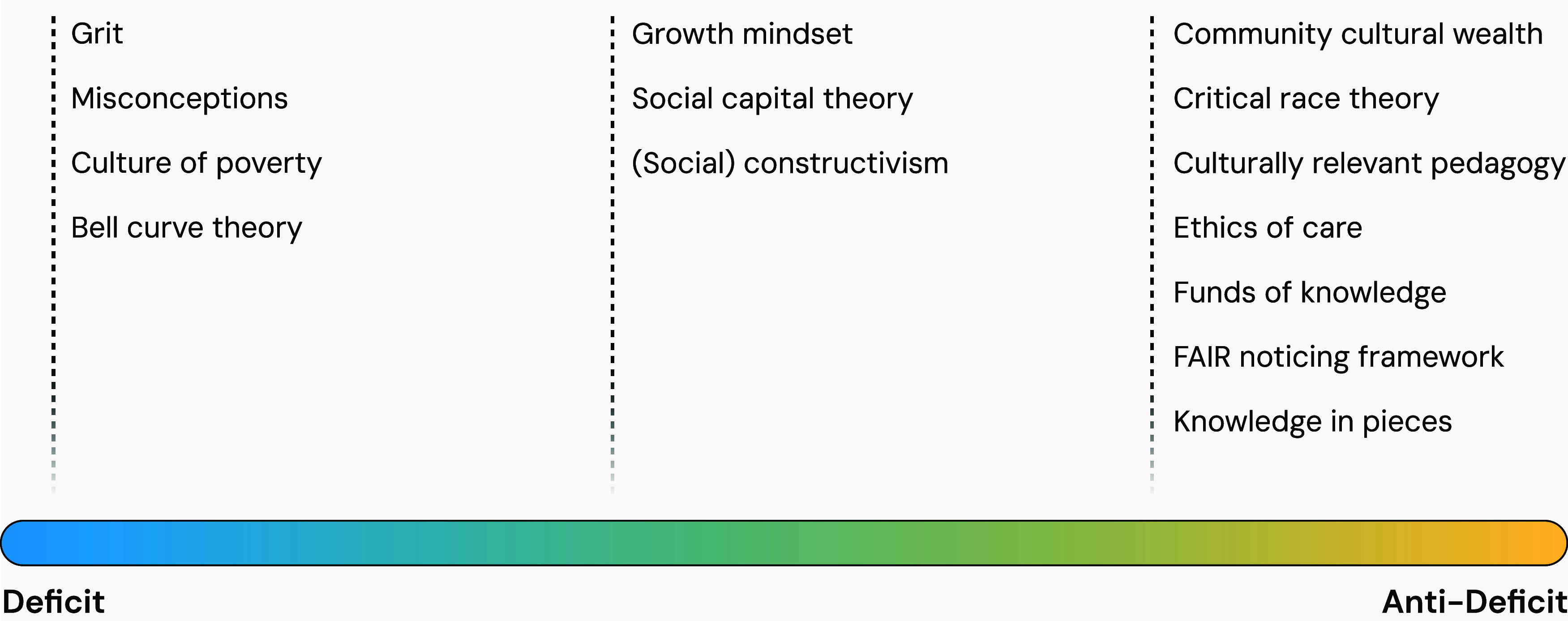
Examples of frameworks indicated by records to promote more anti-deficit- or more deficit-oriented teaching practices or research designs. Importantly, no theory is purely “anti-deficit” or “deficit” but rather works to construct framings that may be more or less likely to be deficit or anti-deficit oriented. For a detailed list, see Supporting Information Table S2.

As shown in Figure 5, some frameworks do not have a clear deficit or anti-deficit orientation. However, based on the impossibility of being “a-deficit,” we highlight that, despite the best of intentions, these frameworks carry the risk of leading stakeholders to “default back” to a deficit-oriented frame. Thus, while a key step, the adoption of “an anti-deficit theoretical framework is necessary but not sufficient to eliminate deficit perspectives”,^59^ which requires conscious and continuous effort.

### Reframing Requires Ongoing Intentionality and Effort at Many Levels

Because of the pervasive, often implicit and unconscious influence of the status quo, and the deficit frames it often primes, reframing is hard and ongoing work for everyone. Because deficit frames are often “unconscious and deeply embedded”,^60^ it is necessary for stakeholders to critically self-reflect on their own beliefs and practices and to develop a “critical consciousness”^89^ of the histories and manifestations of deficit framing in their context, including the ways they and other stakeholders may have unconsciously internalized deficit thinking.

In doing so, it is important to remember that constructing an anti-deficit frame requires more than “shifting the blame” to a new stakeholder, as this still aims to “fix the individual” (albeit a different individual) and fails to identify and address the fundamental causes. In this way, shifting the blame (e.g., from chemistry students to educators) is counterproductive and enables the persistence of systems and structures that negatively impact all stakeholders. Therefore, critical reflection is both something that individual stakeholders must do and something that broader systems and structures must incentivize and support. While in some contexts this may unavoidably begin on the individual level, individuals must work within their sphere of influence to initiate and sustain changes to systems and structures.

It is essential to remember that all stakeholders are unavoidably influenced by their societal context. Stakeholders are not at fault for having internalized elements of deficit-oriented thinking, but they do have the power to decide not to be complicit in perpetuating deficit-oriented frames, systems, and structures and to continuously and actively strive toward anti-deficit reframing. In the context of chemistry education, this operates at many levels. There are some components that chemistry educators have autonomy over in their courses: in addition to critically reflecting on deficit orientations tacitly embedded in course curricula, policies, and activities, postsecondary chemistry educators can recognize that students have likely internalized deficit narratives about themselves. This suggests a need for chemistry educators to consciously work to support students in recognizing their own strengths and ability to succeed in chemistry. For stakeholders with broader spheres of influence, this may also include advocating for and/or instituting systems that structurally support and incentivize educators’ critical reflection and anti-deficit reframing. For example, the American Chemical Society Committee on Professional Training (CPT) “establishes guidelines and standards for the approval of bachelor’s degree programs in chemistry”.^90^ To maintain approval, institutions must submit a periodic report that includes a description of efforts toward inclusive, equitable, and accessible chemistry education. In completing this, departments could examine the ways to support critical reflection and how stakeholders in their programs construct and are impacted by frames.

The evidence discussed here illustrates how anti-deficit reframing is highly context dependent. Thus, one-size-fits-all suggestions of specific “best practices” can be expected to be unproductive. For this reason, we focus here on general mechanisms to empower the reader to consider how these relationships will manifest in their specific context. For detailed examples of this in a variety of contexts, we refer interested readers to existing case studies in this area, such as refs 54, 65, 70, and 91.

### Hold High Expectations, but Make Sure They Matter

A central feature of anti-deficit framing is high expectations. This means holding high expectations for students’ chemistry knowledge and skill development, because they are capable of achieving them. Indeed, “the strongest predictors of learning among students of color are related to faculty who affirm students, maintain high expectations, and provide constructive feedback.”^60^

However, instructors must recognize that deficit-oriented systems have been affecting students for a minimum of nearly two decades before they reach the college chemistry classroom. This has unavoidably impacted students’ prior learning, the expectations students have of themselves, and their beliefs about their own capabilities. However, these same students do have strengths, abilities, and potential. For an anti-deficit reframing, it is essential for instructors to critically reflect on what strengths and abilities are recognized in their classrooms, which strengths and abilities are ignored, and what differential impacts this has on students. Fundamentally, this motivates questions such as those raised by Weatherton and Schussler:^92^ What should “count” as success, and who should have a voice in determining this?

There is considerable evidence that common indicators of success, such as GPA or high-stakes exams, do not accurately indicate students’ knowledge and skills.^93–99^ Currently, GPA and high-stakes exams “matter” because stakeholders (educators, institutions, etc.) construct systems (e.g., program requirements, application review) that make them matter. An anti-deficit reframing would suggest a need to reflect on the extent to which these measures rightly represent what these systems and structures interpret them to represent and, if not, to revise these systems and structures to use higher-quality evidence of knowledge and skills.

In other words, an anti-deficit reframing would suggest a need to re-examine the alignment between metrics and skills that result in academic success and the broader purpose of academic training. For example: chemistry courses commonly rely heavily on individual, high-stakes measures of knowledge and mathematical skills (e.g., solving problems on timed exams) or correct use of formal disciplinary language. One might reflect on the importance of the abilities these measures assess, how accurately and comprehensively they reflect students’ chemistry knowledge and skills, and how relevant they are to students engaged in an ongoing learning process. For example, as an illustrative case:

As I was grading, I realized that although students often did not use the formal words from class, they were still successful in describing these concepts in their own words. An important goal for this class was for students to be able to communicate mathematical ideas, and they were certainly doing that. I imagine that their explanations would be accessible to students who are still learning the mathematics. I, as their instructor, could also understand their explanations and could help in translating their words into more formal mathematical language (ref 65, p. 10)

Similarly, one might ask how aligned these skills are with the actual practice of chemistry beyond the college classroom. As Peck says: testing “narrowly constrains the sorts of knowledge that can be assessed and the means by which it can be displayed. Such tests are individualistic even though problem solving is often collaborative.”^61^ Similar points could be raised about the emphasis on by-hand mathematical computations given the ubiquity of free computational tools, or the emphasis on individual problem solving and the corresponding de-emphasis on oral and written communication of scientific arguments.

There is, of course, an inherent tension here: one must also attend to what disciplinary knowledge and skills are necessary for students to persist in the systems and structures that exist now. This highlights how constructing anti-deficit-oriented frames, systems, and structures is ongoing, multi-level work. Yet, there may be places where educators can diversify the knowledge and skills emphasized in their course, and the ways that students can demonstrate knowledge and skills,^100^ even within broader, deficit-framed systems. There will inevitably be places where stakeholders’ actions are constrained by these systems. Yet: “The only way to dismantle deficit thinking [···] woven into our society, our colleges and universities, and our teaching, is piece by piece.”^70^

## Conclusions

Here, we report a systematic, integrative literature review of the structure and function of deficit and anti-deficit framing. Fundamentally, a stakeholder’s broader context (status quo) primes the frames they construct to understand, interpret, and respond to situations. Given the widespread embeddedness of deficit-oriented narratives within the status quo, explicit and implicit deficit frames are ubiquitous. Deficit framing results in reduced expectations, reduced opportunities, and reduced outcomes for stakeholders as well as psychological and emotional harm. These impacts iteratively reinforce the status quo, cyclically perpetuating the norms, systems, and structures that embed these effects. This self-reinforcing cycle gives momentum to existing deficit frames, such that anti-deficit framing requires intentional and sustained effort.

There are tangible ways that deficit framing functions to perpetuate existing inequities in postsecondary chemistry education. Due to the entrenched nature of deficit framing in society, and the impossibility of situations to be decontextualized or “frame-less,” it is impossible to be “a-deficit.” Trying to be neutral, or not attending to deficit and anti-deficit framing, will “default” back to existing societally-imposed deficit frames. Furthermore, reframing efforts often fall victim to “deficit creep,” where deficit-oriented attributes sneak back into efforts to improve equity, inclusivity, and social justice. Similarly, we highlight and caution against “frame-shifting,” where a deficit frame is redirected to place blame on a different stakeholder but still fundamentally leaves unexamined and intact the underlying systems, structures, and beliefs that resulted in a need for blame.

In this way, a rigorous, equitable, and inclusive chemistry education requires intentional reframing: sustained, concerted efforts to construct and maintain anti-deficit frames in chemistry teaching, mentoring, professional development, and education research. This requires efforts at the individual, institutional, and societal levels. Thus, approaches to enacting and sustaining these changes are an important area for continued focus and research. This is hard and long-term work, but it fundamentally works to uphold high expectations, improve chemistry education, and advance the progress of science. It is not possible to be neutral: either our actions will serve to support deficit-oriented systems that ultimately harm science, scientists, educators, and students, or we can work toward anti-deficit reframing to better support scientific progress and chemistry education.

## Supplementary Material



## Notes

Analyzed records; (anti-)­deficit priming frameworks, theories, and pedagogies; and triangulation between analyzed records and external data sources (PDF)
