# Supplementary material for: How Perspective Directs Outcomes: The Structure and Function of Deficit and Anti-deficit Framing and Their Relevance to Chemistry Education
Source: JACS Au. 2026 Apr 17;6(5):2780–97. doi: 10.1021/jacsau.6c00081 (PMC13213517; doi:10.1021/jacsau.6c00081)
Supplement: Supplementary file 1 [file au6c00081_si_001.pdf]

# *How Perspective Directs Outcomes: The Structure and Function of Deficit and Anti-Deficit Framing and their Relevance to Chemistry Education*

*Elizabeth B. Vaughan, Josephine Bicknell, Henry Holleb, Kodinna Anachebe, Tia Kledzik, Nicole M. James**

*Department of Chemistry, Reed College, Portland, OR 97202, USA.*

** Corresponding author: njames@reed.edu*

**Supporting Information Table of Contents**

Included Records.....…………………………………………………………………………2

(Anti-)Deficit Priming Frameworks, Theories, and Pedagogies …………………………………5

Triangulation Between Analyzed Records and External Data Sources …………………………..7

**Included Records**

Table S1 reports the title, author(s), publication year, version date (i.e., date listed on the version of each document referenced in this literature review, which may differ for advance online versions), journal, and country of origin for each of the 29 records analyzed in this literature review.

**TABLE S1.** Included Records

| Title | Author(s) | Year | Version Date | Journal | Country |
|---|---|---|---|---|---|
| Beyond deficit thinking: Providing access for gifted african american students | Ford, D.Y.; Harris, J.J.; Tyson, C.A.; Trotman, M.F. | 2001 | 2001 | Roeper Review | United States |
| Displacing Deficit Thinking in School District Leadership | Skrla, L.; Scheurich, J. J. | 2001 | 2001 | Education and Urban Society | United States |
| Providing Access for Culturally Diverse Gifted Students: From Deficit to Dynamic Thinking | Ford, D. Y.; Grantham, T. C. | 2003 | 2003 | Theory into Practice | United States |
| Challenging Deficit Thinking | Weiner, L. | 2006 | 2006 | Educational Leadership | United States |
| The Miseducation of a Beginning Teacher: One Educator's Critical Reflections on the Functions and Power of Deficit Narratives | Pollack, T. M. | 2012 | 2012 | Multicultural Perspectives | United States |
| Meritocracy, Deficit Thinking and the Invisibility of the System: Discourses on Educational Success and Failure | Clycq, N.; Nouwen, M. A. W.; Vandenbroucke, A. | 2014 | 2014 | British Educational Research Journal | Belgium |
| Educator Beliefs and Cultural Knowledge: Implications for School Improvement Efforts | Nelson, S. W.; Guerra, P. L. | 2014 | 2014 | Educational Administration Quarterly | United States |
| What Latino Students Want from School | Irizarry, J. G. | 2015 | 2015 | Educational Leadership | United States |
| "Low Income Doesn't Mean Stupid and Destined for Failure": Challenging the Deficit Discourse around Students from Low SES Backgrounds in Higher Education | McKay, J.; Devlin, M. | 2016 | 2015 | International Journal of Inclusive Education | Australia |
| "Really Just Lip Service": Talking about Diversity in Suburban Schools | Tyler, A. C. | 2016 | 2016 | Peabody Journal of Education | United States |
| Deficit Discourse and Labeling in | Garcia-Olp, M.; | 2017 | 2017 | Journal of | United |

| | | | | | |
|---|---|---|---|---|---|
| Elementary Mathematics Classrooms | Van Ooyik, J.; Kitchen, R. | | | Mathematics Education at Teachers College | States |
| The Presence of Deficit Thinking among Social Studies Educators | Keefer, N. | 2017 | 2017 | Journal of Social Studies Education Research | United States |
| Re-Conceptualizing Student Success in Higher Education: Reflections from Graduate Student Affairs Educators Using Anti-Deficit Achievement Framework | Pérez II, D..; Ashlee, K. C.; Do, V. H.; Karikari, S. N.; Sim, C. | 2017 | 2017 | Journal on Excellence in College Teaching | United States |
| "We Can't Fix That": Deficit Thinking and the Exoneration of Educator Responsibility for Teaching Students Placed at a Disciplinary Alternative School | Kennedy, B. L.; Soutullo, O. | 2018 | 2018 | Journal of At-Risk Issues | United States |
| Seeping Deficit Thinking Assumptions Maintain the Neoliberal Education Agenda: Exploring Three Conceptual Frameworks of Deficit Thinking in Inner-City Schools | Sharma, M. | 2018 | 2018 | Education and Urban Society | Canada |
| Anti-Deficit Narratives: Engaging the Politics of Research on Mathematical Sense Making | Adiredja, A. P. | 2019 | 2019 | Journal for Research in Mathematics Education | United States |
| Everyday Examples about Basis from Students: An Anti-Deficit Approach in the Classroom | Adiredja, A. P.; Bélanger-Rioux, R.; Zandieh, M. | 2020 | 2019 | PRIMUS: Problems, Resources, and Issues in Mathematics Undergraduate Studies | United States |
| Anti-Deficit Framing of Sociological Physics Education Research | Exarhos, S. | 2020 | 2020 | Physics Teacher | United States |
| Academic English as Standard Language Ideology: A Renewed Research Agenda for Asset-Based Language Education | MacSwan, J. | 2020 | 2020 | Language Teaching Research | United States |
| Understanding First-Generation Undergraduate Engineering Students' Entry and Persistence through Social Capital Theory | Martin, J. P.; Stefl, S. K.; Cain, L. W.; Pfirman, A. L. | 2020 | 2020 | International Journal of STEM Education | United States |
| The Problem with Grit: Dismantling Deficit Thinking in Library Instruction | Tewell, E. | 2020 | 2020 | portal: Libraries and the Academy | United States |

| | | | | | |
|---|---|---|---|---|---|
| Teachers' understanding of racial inequity predicts their perceptions of students' behaviors | Legette, K. B.; Halberstadt, A. G.; Majors, A. T. | 2021 | 2021 | Contemporary Educational Psychology | United States |
| Teacher Noticing from a Sociopolitical Perspective: The FAIR Framework for Anti-Deficit Noticing | Louie, N.; Adiredja, A. P.; Jessup, N. | 2021 | 2021 | ZDM: Mathematics Education | United States |
| Towards Anti-Deficit Education in Undergraduate Mathematics Education: How Deficit Perspectives Work to Structure Inequality and What Can Be Done about It | Peck, F. | 2021 | 2020 | PRIMUS: Problems, Resources, and Issues in Mathematics Undergraduate Studies | United States |
| Improving the well-being of learners with visual impairments in rural Lesotho schools: An asset-based approach | Ramatea, M. A.; Khanare, F. P. | 2021 | 2021 | International Journal of Qualitative Studies on Health and Well-being | Lesotho |
| "They Don't Know What They Need": A Call for Critical Reflection in Community School Leadership to Address the Pervasive Creep of Deficit Perspectives | Lewis-Durham, T.; Saastamoinen, M. | 2022 | 2022 | Journal of Cases in Educational Leadership | United States |
| Reframing Educational Outcomes: Moving beyond Achievement Gaps | Shukla, S.Y.; Theobald, E.J.; Abraham, J.K.; Price, R.M. | 2022 | 2022 | CBE Life Sciences Education | United States |
| 'My abilities were pretty mediocre': Challenging deficit discourses in expanding higher education systems | Ávila Reyes, N.; Navarro, F.; Tapia-Ladino, M. | 2023 | 2021 | Journal of Diversity in Higher Education | Chile |
| Community, collaboration, and climate | Hardin, J.; Shahriari, S. | 2023 | 2022 | PRIMUS: Problems, Resources, and Issues in Mathematics Undergraduate Studies | United States |

## (Anti-)Deficit Priming Frameworks, Theories, and Pedagogies

Table S2 reports frameworks, theories, and pedagogies that are described by the analyzed records to prime a deficit, anti-deficit, or ambiguous frame.

**Table S2.** (Anti-)Deficit Priming Frameworks, Theories, and Pedagogies

| Primed Frame | Frameworks, Theories, and Pedagogies | Records |
|---|---|---|
| Deficit | Bell Curve Theory | Sharma, 2018 |
| | Culture of Poverty | Tyler, 2016; Keefer, 2017 |
| | Describe, Explain, Predict, Prescribe | Sharma, 2018; Garcia-Olp et al., 2017 |
| | Expert-Novice Comparisons | Adiredja, 2019 |
| | Initiate, Respond, Evaluate | Louie et al., 2021 |
| | Misconceptions Research | Adiredja, 2019 |
| | Structuralism/Positivism (In Science) | Sharma, 2018 |
| | Subtractive Assimilation/ Subtractive Schooling | Sharma, 2018 |
| Ambiguous | (Social) Constructivism | Adiredja, 2019; Martin et al., 2020 |
| | Cultural Capital Framework | Ávila Reyes et al., 2021 |
| | Growth Mindset | Tewell, 2020 |
| | Social Capital Theory | Sharma, 2018; Martin et al., 2020 |
| | Student Identity/Mathematical Identity | Garcia-Olp et al., 2017 |
| Anti-Deficit | Anti-Deficit Achievement Framework | Pérez II etal., 2017 |
| | Anti-Racist Pedagogy | Pollack, 2012 |
| | Caring Theory | Sharma, 2018 |
| | Community Cultural Wealth | Pérez II etal., 2017; Ávila Reyes et al., 2021; Shukla et al., 2022 |
| | Community Equity Literacy | Lewis-Durham & Saastamoinen, 2022 |
| | Critical Consciousness | Keefer, 2017 |
| | Critical Information Literacy | Tewell, 2020 |
| | Critical Race Theory | Tyler, 2016; Pérez II etal., 2017; Adiredja, 2019; Tewell, 2020 |
| | Culturally Engaging Campus Environments Model | Pérez II etal., 2017 |
| | Culturally Sustaining/Relevant/Responsive Pedagogy/Teaching | Keefer, 2017; Tewell, 2020 |
| | Equity Pedagogy | Keefer, 2017 |

| | | |
|---|---|---|
| | Ethics of Care | Shukla et al., 2022 |
| | Framing, Attending, Interpreting, and Responding Framework | Louie et al., 2021 |
| | Funds of Knowledge | Nelson and Guerra, 2014; Keefer, 2017; Adiredja, 2019; Tewell, 2020; MacSwan, 2020; Ávila Reyes et al., 2021 |
| | Inquiry Based Pedagogy | Keefer, 2017 |
| | Knowledge in Pieces | Adiredja, 2019 |
| | LatCrit Theory | Adiredja, 2019 |
| | Mathematics for Social Justice | Adiredja, 2019 |
| | Multicultural Education | Pollack, 2012 |
| | Post-Structuralism | Adiredja, 2019 |
| | Social Justice Theory | Keefer, 2017 |
| | Sociopolitical Framework/Perspectives | Garcia-Olp et al., 2017; Adiredja, 2019 |
| | Structural Inequality Framework | Ávila Reyes et al., 2021 |
| | The Algebra Project | Adiredja, 2019 |
| | Theory of Multiple Intelligences | Ford et al., 2001; Ford & Grantham, 2003 |
| | Thriving Quotient | Pérez II etal., 2017 |
| | Triarchic Theory of Intelligence | Ford et al., 2001; Ford & Grantham, 2003 |

**Triangulation Between Analyzed Records and External Data Sources**

One method of triangulation used in this study was the comparison of findings between the analyzed records and external data sources (N=14). External data sources included relevant texts referenced frequently within the analyzed records (n=2), additional texts that the research team believe to be related to the findings (n=2), and a selection of peer-reviewed academic scholarship published after our record screening process (n=10). All external data sources were reviewed for disconfirming evidence by one or more member(s) of the research team (N.M.J, E.B.V, and/or T.K.). No disconfirming evidence was found, and considerable confirming evidence was found, as summarized in Table S3.

**Table S3.** Alignment of results between analyzed records and external data sources; full reference information can be found on SI page 22

| | **External Data Source Reference Number** | | | | | | | | | | | | | |
|---|---|---|---|---|---|---|---|---|---|---|---|---|---|---|
| **Topic** | 1 | 2 | 3 | 4 | 5 | 6 | 7 | 8 | 9 | 10 | 11 | 12 | 13 | 14 |
| Deficit and anti-deficit frames | | | | | | | | | | | | | | |
| Deficit frame | x | x | x | x | x | x | x | | x | x | x | x | x | x |
| Anti-deficit frame | x | x | x | | x | x | x | | x | x | x | x | x | x |
| Frames are neither binary, nor static | | | x | x | | | | x | | | x | | x | x |
| Deficit framing: impacts on stakeholders | | | | | | | | | | | | | | |
| Reduced expectations | x | | | | | x | x | x | | x | | | x | x |
| Absolving responsibility and denying agency | | | | x | | x | x | x | | x | | | x | x |
| Over-disciplining and overlooking | x | x | x | x | x | x | x | x | | x | | | x | x |
| Reduced performance or outcomes | x | | | | | x | x | x | | x | | | x | x |
| Internalization | x | x | | | | x | | x | | x | | | x | x |
| Fixing the individual | x | | x | x | | x | | x | | | x | | x | x |
| Assimilation pressure | x | x | x | | | x | x | x | | x | x | | x | x |
| Epistemic violence and cultural violence | x | x | x | x | | x | | x | x | x | | x | x | x |
| Psychological and emotional harm | | x | x | x | | x | x | x | | | | x | x | x |

| | | | | | | | | | | | | | | |
|---|---|---|---|---|---|---|---|---|---|---|---|---|---|---|
| Anti-deficit framing: Impacts on stakeholders | | | | | | | | | | | | | | |
| High expectations | x | x | | x | | | | | | x | | | x | x |
| Improved performance or outcomes | x | | | x | | | | | | | | | x | x |
| Recognizing multiple ways of knowing and succeeding | x | x | | x | x | x | x | | | x | | | x | x |
| Status quo: Societally-imposed deficit frames | | | | | | | | | | | | | | |
| Deficit talk and deficit noticing | x | x | x | x | | | x | x | x | x | x | | x | x |
| Partial or biased data | | | x | | x | | x | x | | x | | | x | x |
| Individualism and the myth of meritocracy | | x | x | x | | | x | x | | | x | x | x | x |
| White supremacy | x | x | x | x | | x | x | x | x | x | x | | x | x |
| Racism | x | x | x | x | x | x | x | x | x | x | x | | x | x |
| Stereotypes | x | | x | x | x | | x | x | | x | | | x | x |
| Socioeconomic status | | x | x | x | x | x | x | x | x | x | | | x | x |
| Anti-deficit framing: Disrupting deficit frames | | | | | | | | | | | | | | |
| Critical reflection and critical consciousness | x | x | | x | | x | x | x | x | x | | | x | x |
| Recognizing stakeholder assets and belief in stakeholders' abilities | x | x | | x | x | x | x | | | x | | | x | x |
| Compassion and empathy | x | x | | x | | | | | | x | | | x | x |
| (Multi-)Cultural competence | x | x | | x | | x | | | x | x | | | x | x |
| Data literacy | | | | x | | x | | x | | | | | x | x |
| High-quality training and structural support | x | x | | x | | x | x | | x | | | x | x | x |
| Ongoing reflective vigilance | x | x | | x | | x | | | | x | | | x | x |

### *Deficit and anti-deficit frames*

Within the external data sources, **deficit frames** are described as follows:

> “Deficit thinking is an ideological phenomenon which frames marginalised individuals as deficient and in need of corrective interventions. It is a victim-blaming, person-centred narrative which locates faults within marginalised individuals and deflects attention away from broader socioeconomic structures of discrimination and injustice” (Cushing, 2025, p. 3)

> “Deficit perspectives posit that any existing disparities in educational outcomes in STEM for Latine students are a product of the students’ lack of interest in STEM fields, poor academic preparation and/or motivation, among other ‘faults.’” (Hernandez Negrete et al., 2023, p. 1)

These descriptions align with those seen in the analyzed records, and support the notion that a deficit frame presupposes that failure or perceived deficits result from intrinsic, inherent characteristics of a stakeholder or their background. Additionally, these descriptions highlight the ways in which a deficit frame holds an individual stakeholder responsible for deficiencies that are located in social norms, structures, and systems.

Alternatively, **anti-deficit frames** are described as follows:

> “Anti-deficit school leaders believe that all students are capable of achievement and that no educator should limit students’ aspirations based on the educator’s ignorance or personal biases.” (Lowery and Romero-Johnson, 2025, p. 541)

> “The importance of advancing anti-deficit perspectives is they shift the focus from the individual level (i.e., assuming that any failure to meet metrics or standards are the fault of the person) to the cultural and structural level (i.e., explicating how educators can reimagine systems and practices to better meet the needs of minoritized communities” (Pérez II et al., 2025, p. 640)

These descriptions once again align with analyzed records and suggest that an anti-deficit frame presupposes that a stakeholder is capable of success. Additionally, anti-deficit framing attributes failure or perceived deficits to insufficiencies or inequities in systems and structures.

In both the analyzed records and the external data sources, deficit and anti-deficit frames are described as **neither binary, nor static**. For example, one external data source presents:

> “[..] two important shifts in practice that surfaced in the analysis of Douglas’ pedagogical journey [..]: 1) from a deficit perspective of student behavior to an asset based stance focused on student agency and leadership and 2) from a

> curriculum centered on dominant culture to a culturally relevant and sustaining curriculum. (Friedman, 2024, p. 769)

*Deficit framing: impacts on stakeholders*

The external data sources used for triangulation described the same impacts of deficit framing on stakeholders as the analyzed records, including:

**Reduced expectations** for stakeholders**,**

> "If researchers continue to approach these environments believing in their inherent depravity, the risk of perpetuating deficit ideologies that are grounded in the low expectations for school environments and teacher excellence will remain." (Holtz et al., 2024, p. 534)

> "Deficit thinking is rooted in cultural deprivation theory, which pathologizes, or identifies cultural inadequacies such as delinquent behavior, low expectations, and lack of motivation as the root of the problem for historically marginalized people" (Lowery and Romero-Johnson, 2025, p. 528-529)

**Absolving responsibility** and **denying agency** of stakeholders,

> In this manner, this deficit approach absolves educators, educational institutions, administrators, and researchers from any responsibility in mediating the disparate outcomes and negates the roles that outdated pedagogical practices, structural racism, discrimination and disciplinary bias have in limiting Latine students' success in STEM. (Hernandez Negrete et al., 2023, p. 1)

> Victim blaming avoids the structural inequalities present in Black and Brown students' schooling location and contributes to the oppressive nature of the deficit thinking model. It is the oppressors (teachers, administrators, school boards, or other government officials) who hold power over communities of color. The mismatch between oppressor and student fails to address the possibilities of school success and victim blaming persists. (Holtz et al., 2024, p. 523)

**Over-disciplining** and **overlooking** stakeholders,

> "Academics, activists, and abolitionist organisations have shown how this kind of deficit thinking contributes to the so-called 'discipline gap' – by which racialised

and low-income children are more likely to be perceived as disruptive and receive punishments." (Cushing, 2025, p. 4)

Contrary to popular belief, the highest rates of disproportionate discipline practices amongst Black and Brown students continue to be in predominantly White, wealthy suburban schools [...] And, while these schools provide greater access to course offerings, resources, and extracurricular activities, Black and Brown students identified a multitude of racial disparities including an underrepresentation in advanced courses, feelings of isolation, and inaccessible after school programs due to high costs or lack of transportation (Holtz et al., 2024, p. 520)

**Reduced performance** or **outcomes** for stakeholders,

"In fact, teachers' deficit beliefs about students and their families may contribute to the persistence of inequitable educational outcomes, despite ongoing reform efforts" (Benedict-Chambers et al., 2025, p. 2)

"Deficit thinking enacted by school leaders reproduces inequitable outcomes on traditional measures such as tests or graduation rates." (Lowery and Romero-Johnson, 2025, p. 529)

Stakeholders' **internalization** of deficit frames,

"Deficit mind-sets affect how educators treat students and often contribute to the development of self-perceptions of inferiority among students [...] and school leaders' attempts to explain away educational inequities as "normal and inevitable" (Lowery and Romero-Johnson, 2025, p. 529)

"Internalisation of deficit perspectives are visible (and audible) in research narratives where English language learners describe themselves as inferior, stupid, and illegitimate in social interactions that employ English discourses" (Cabiles, 2024, p. 4)

"I thought I was a subpar student and was bombarded by messages-from Black people, White people, the media–that told me that the reason was rooted in my race…which made me more discouraged and less motivated as a student…which only further reinforced for me the racist idea that Black people just weren't very studious…which made me feel even more despair or indifference" (Kendi, 2023 p. 6) (full quote provided; ellipses are present in text)

A focus on **fixing the individual** (stakeholder),

> "In their first semester, they [preservice teachers] either did not attend to or held a deficit view of content, framing students' ideas as needing to be fixed rather than as valuable pieces of sensemaking." (Benedict-Chambers et al., 2025, p. 6)

> "The paradigm that student behavior should conform to teacher expectations and that disruptions or opposition are the fault of the student is widely held. Douglas assumed the solution involved fixing the student" (Friedman, 2024, p. 772)

> "Americans have long been trained to see the deficiencies of people rather than policy. It's a pretty easy mistake to make: People are in our faces. Policies are distant. We are particularly poor at seeing the policies lurking behind the struggles of people" (Kendi, 2023, 34)

Pressure on stakeholders to **assimilate**,

> "From a deficit approach, the pupils, students or their families need to change or assimilate to the dominant norms, values and conventions." (Mampaey and Huisman, 2022, p. 1234)

> "Deficit approaches in classrooms often carry racial, class, and heterosexual assumptions of family that signal to students that they must adopt White middle class familial practices and roles in order to be successful" (Hernandez Negrete, 2023 et al., p. 8)

> "Assimilationist ideas position any racial group as the superior standard that another racial group should be measuring themselves against, the benchmark that they should be trying to reach. Assimilationist ideas typically position White people as the superior standard" (Kendi, 2023, p. 36)

**Epistemic violence** and **cultural violence** toward stakeholders,

> "Deficit approaches operate from the assumption that Latine students lack access to networks and community resources (i.e., social capital) essential for their academic progression" (Hernandez Negrete et al., 2023, p. 9)

> "Numerous accounts of European colonial schooling from the 1600s onwards demonstrate how Indigenous children were subjected to physical and psychological abuse by teachers if they were heard to be using their own language [...] At the core

> of these deficit logics is the narrative that marginalised communities can be remediated through education, with schooling providing them with the standards that their homes allegedly lack." (Cushing, 2025, p. 5)

And **psychological and emotional harm** to stakeholders,

> "The classroom can feel like an isolating place for students of Color and other minoritized students, especially at predominantly White institutions. Students may feel invisible or hypervisible [among] their peers and professors due to their race, socioeconomic status, ability, sexual orientation, religion or gender expression." (Pérez II et al., 2025, p. 647)

> "Even more concerning is the effect that this deficit paradigm has on the parents within the community. The exclusionary practices on Black and Brown students extended to their families with parents reporting feelings of fear as their young children entered school." (Holtz et al., 2024, p. 520)

*Anti-deficit framing: Impacts on stakeholders*

Similarly, the external data sources used for triangulation described the same impacts of anti-deficit framing on stakeholders as the analyzed records, including:

Holding **high expectations** for stakeholders,

> "Leaders who uphold high expectations for Latina/o/x students dismantle deficit thinking because they do not allow low expectations to frame their actions. One result of high expectations is equitable representation of Latina/o/x students in rigorous academic opportunities." (Lowery and Romero-Johnson, 2025, p. 536)

> "Because Douglas believed that student-led learning could be made accessible for all students, his change in perspective led to higher expectations for all students and not just students in one particular class or neighborhood. [...] Calling out the deficit perspective that many people hold about students living in poverty demonstrates Douglas' progress toward antiracism as he moved from acting on his own unconscious deficit view to recognizing how that view lived in others. Douglas came to understand that the ways he reacted to student behavior, the ways he structured the learning environment, and the curriculum he chose had a powerful influence on student outcomes. [...] Ultimately, Douglas moved from believing a

student needed to change, to changing his practice, and ultimately transforming his expectations of what students can achieve.” (Friedman, 2024, p. 774-775)

**Improved performance or outcomes** for stakeholders**,**

“The more leadership Douglas offered the students, the better they performed and the higher his expectations grew for their leadership and academic achievement. He described their accelerated academic growth:
‘They built the skills and that translated into their scores. The year before we had 10 percent passing on their math state exams. Then we did this type of work and that same group, they went up to 50 percent passing. There was no magic pill aside from them being empowered to lead their own instruction, and that made them more eager to come to school. So attendance went up, everyone participated more, remained engaged, gave and got feedback.’” (Friedman, 2024, p. 774)

“Students will strive to be competent when the knowledge and skills they are developing is organized for important problem solving. Students with alleged deficits when encouraged to become competent, respond accordingly.” (Valencia, 1997, p. 231)

And **recognizing multiple ways of knowing and succeeding,**

“[...] teachers who engage in anti-deficit noticing frame students as “full human beings with many resources” [...]. They attend to students as distinct individuals with their own personalities, participation styles, life experiences, and funds of knowledge [...]. They interpret students’ characteristics—personalities, participation styles, life experiences—as valuable resources. And they respond by creating an environment that invites students to share their ideas and uses their knowledge and experiences to enhance their learning and that of their peers” (Benedict-Chambers et al., 2025, p. 3)

“Latine resistant capital refers to the wisdom, knowledge, and information Latine students have that helps them combat systems of oppression [...]. Deficit approaches assume Latine students do not possess this knowledge of resistance to challenge inequality. This capital takes a non-deficit approach by highlighting the intergenerational wisdom passed onto Latine students from their families to resist the status quo” (Hernandez Negrete et al., 2023, p. 6)

*Societally-imposed deficit frames*
The norms and status quo described to impact framing were the same in the analyzed records and external data sources. The external data sources described:

**Deficit talk** and **deficit noticing**,

> "These discourses contribute to "deficit noticing," where teachers, even those well-intentioned, may unintentionally perceive and interpret students' behaviors in ways that reinforce stereotypes, ultimately hindering equitable learning opportunities." (Benedict-Chambers et al., 2025, p. 2)

> "…when PSTs [preservice teachers] in the Deficit-Framed Diversity profile discussed language acquisition, they frequently referred to relationships between low socioeconomic status and low vocabulary acquisition, harkening to research on word gaps: 'Children of poverty backgrounds often come into school with low-vocabulary exposure than their white/higher social-econ status' (Post 101). PSTs in this profile acknowledged that diversity does matter, but they seemed to highlight the challenges posed by diversity." (Kwok et al., 2023, p. 59-60)

(Over-)Reliance on **partial or biased data**,

> "Researchers who maintain deficit thinking contribute to a pseudoscience that is entrenched with negative attitudes toward Black and Brown students, flawed methodologies, and findings that uphold deficit paradigms surrounding school location. This pseudoscience is further exacerbated by the temporal changes in society wherein the alleged deficits are attributed to inferior genetic makeup, lesser culture and class, or insufficient familial constructs." (Holtz et al., 2024, p. 523)

> "These colonial logics continue to shape contemporary schools, where children are subjected to tests, curricula, pedagogies and policies designed to systematically exclude deviations from idealised norms along the intersections of race, class and ability" (Cushing, 2025, p. 5)

> "It [a GRE prep course] revealed the bait and switch at the heart of standardized tests–the exact thing that made them unfair: She [the instructor] was teaching test-taking form for standardized exams that purportedly measured intellectual strength. My classmates and I would get higher scores–two hundred points, as promised–than poorer students, who might be equivalent in intellectual strength but did not

have the resources or, in some cases, even the awareness to acquire better form through high-priced prep courses" (Kendi, 2023, pp 112-113

**Individualism** and the **myth of meritocracy**

In a context with widespread beliefs in deficit (and meritocratic) ideology, where powerful social actors (e.g. elite, white men) also benefit from this ideology, these actors may truly belief in equal opportunities. Acknowledgement of unfair bias in the system would be a threat to their own self-esteem. That would imply that they have to acknowledge that their own powerful social positions are the result of unfair bias instead of intelligence or skills. (Mampaey and Huisman, 2022, p. 1264)

"Because of the way the human mind works–the so-called 'attribution effect,' which drives us to take personal credit for any success–those of us who prepped for the test would score higher and then walk into better opportunities thinking it was all about us: that we were better and smarter than the rest and we even had inarguable, quantifiable proof. Look at our scores!" (Kendi, 2023, p. 113)

"'Some scholars would have us believe that educability is largely dependent on individual intellectual ability and that social, political, and economic conditions within the schools and society are largely unrelated to "why some of our children are so much more educable than others"" (Valencia, 1997, p. 8; *Quoting* Valencia and Aburto, 1991 and Hawkins, 1984)

**White supremacy**,

"…he was aware of biased messages from adults who used coded language to warn white students not to socialize with students of color outside of school or travel to the parts of town where they lived. The conflict between the "culture of collegiality amongst the student body created at school" and the assumption of superiority from white adults in his life stayed with him." (Friedman, 2024, p. 770)

"At the core of these critiques is the idea that schools reward behaviours encoded as white, able-bodied and middle-class – or what Youdell (2006) conceptualises as the ideal learner" (Cushing, 2025, p. 3)

"[...] Fracis Galton–a half cousin of Charles Dawin–hypothesized in *Hereditary Genius* that the 'average intellectual standard of the negro race is some two grades

> below our own.' [...] Alfred Binet and Theodore Sion [...] developed an IQ test in 1905 that Stanford psychologist Lewis Terman revised and delivered to Americans in 1916. These 'experimental' tests would show 'enormously significant racial differences in general intelligence, differences which cannot be wiped out by any scheme of mental culture' " (Kendi, 2023, p 114)

**Racism** and racial and ethnic **stereotypes,**

> "In the context of students considered as 'cultural minorities', schooling can perpetuate deficit thinking through entrenched systems and practices that are often racist in their genesis [...] the cultures and languages of students from African American and native American backgrounds are perceived as liabilities and problems when measured against the standard dominant (and powerful) culture and language (i.e., English)." (Cabiles, 2024, p. 3).

> "Within the STEM classroom, these deficit perspectives underpin traditional pedagogies that promote highly competitive 'survival of the fittest' mentalities that see education as a tool to weed out the 'weak' [...] These perspectives disadvantage Latine students within this competitive culture because they perpetuate racialized messages that construe Latine students as 'failing,' 'weak,' 'in need of help,' and 'deficient,' while their White or more privileged peers are viewed as 'successful,' 'strong,' and 'capable.'" (Hernandez Negrete et al., 2023, p. 3)

> "The acceptance of an academic-achievement gap is just the latest method of reinforcing the old racist idea: Black intellectual inferiority. [...] implicit in this idea is that academic 'achievement' can only be measured by statistical instruments like test scores and dropout rates. There is an even more sinister implication in achievement-gap talk–that disparities in standardized test scores accurately reflect disparities in intelligence among racial groups." (Kendi, 2023, p. 113)

And bias based on **socioeconomic status**,

> "Yet these narratives rely on deficit thinking, which begins from the assumption that the homes of marginalised children are devoid of routines and boundaries – and so school is a place where they are socialised into what they allegedly lack. Whilst such deficit ideologies about the parenting styles of low-income families are nothing new, they have seen a marked resurgence as part of the academies agenda." (Cushing, 2025, p. 16)

"When Douglas interviewed for an administrative position in a suburban district, the superintendent questioned his career trajectory. "They looked at my resume and they were impressed, but then the superintendent went, 'Hmm, you did it backwards,' meaning I went from a high performing predominantly white district to the opposite, and it just showed me that people aren't as far along as we want to believe." Douglas understood "backwards" as coded language which belied the assumption that given a choice everyone would choose to teach in affluent white schools. Further, he recognized the deficit perspective implicit in the idea that teaching in a marginalized neighborhood is the purview of less qualified teachers." (Friedman, 2024, p. 778)

*Anti-deficit framing: Disrupting deficit frames*

Finally, the external data sources used for triangulation described the same tools for challenging or disrupting existing societally-imposed deficit frames in an effort to employ anti-deficit frames. The external data sources describe:

Engaging in **critical reflection** and developing **critical consciousness,**

"Without critical examination of the traditional ideologies, perspectives and biases that have shaped STEM pedagogies and practices, STEM educators can unknowingly employ and reproduce deficit-based interactions (lowered expectations, microaggressions) when working with Latine students" (Hernandez Negrete et al., 2023, p. 3)

"Given the complexity of teaching and the pervasive influence of power and culture in classrooms, it is essential that teachers develop the ability to notice for equity [...] in explicitly anti-deficit ways [...]. Noticing for equity requires teachers to actively attend, interpret, and respond to aspects of classroom life that cultivate equitable learning environments." (Benedict-Chambers et al., 2025, p. 1)

**Recognizing stakeholder assets** and affirming their **belief in stakeholders' abilities,**

"One way school leaders can address deficit views among educators is to reframe thinking about deficits to recognizing the strengths, assets, or funds of knowledge [...] that students and families bring to school that have not been recognized but can be incorporated into school educational practices" (Lowery and Romero-Johnson, 2025, p. 529)

“Rather than displaying an internalised deficit thinking about their own (and others’) cultural resources, the narratives of the students demonstrate strengths-based understandings and utilisation of these resources. Evidently, students can position themselves through a strengths-based framework and locate themselves from a perspective of resource-full participants in the learning environment.” (Cabiles, 2024, p. 13)

“What if different environments lead to different kinds of achievement rather than different levels of achievement? What if the intellect of a low-testing Black child in an impoverished Black school is different from–not inferior to–the intellect of a high-testing White child in a rich White school? What if we measured intelligence by how knowledgeable individuals are about their own environments? What if we measured intellect by an individual’s desire to know? What if we realized the best way to ensure an effective educational system is not by standardizing our curricula and tests but by standardizing the opportunities available to all students?” (Kendi, 2023, p 115)

Engaging in **compassion** and **empathy,**

“Noticing for equity in anti-deficit ways requires framing student teacher and peer relationships and interactions as integral to learning. Attending to the relational dimensions of teaching allows educators to see strong, egalitarian bonds as essential resources for learning. By prioritizing these connections, teachers can create environments that empower students, especially those from marginalized backgrounds who may have experienced negative educational encounters [...]. Positive interactions foster greater engagement, achievement, and a sense of belonging. By valuing peer relationships, teachers can cultivate collaborative spaces where students feel safe to share ideas and engage with one another” (Benedict-Chambers et al., 2025, p. 3)

“Cecilia explained, “I try to understand what [families’] needs are, where they’re coming from, so that we can better serve them.” She added, “To me, it’s not you hold a leadership position, and you are in charge.” An anti-deficit approach meant that school leaders worked alongside parents rather than as experts who assumed parents needed to be told what to do.” (Lowery and Romero-Johnson, 2025, p. 539)

Developing **(multi-)cultural competence,**

> “Students’ ‘cultural competence’ is identified as one of the key principles of CRPs where students are given opportunities to re-define the existing curriculum, demonstrate their funds of knowledge, and co-facilitate building relationships between schools and their communities” (Cabiles, 2024, p. 14)

> “[...] the Cultural Competence profile [...] involves maintaining students’ ‘cultural integrity’ or using their ‘culture as a vehicle for learning’ alongside supporting their academic success [...] Within this profile, [preservice teachers] articulated cultural competence as providing diverse representation in curricular materials and connecting learners’ cultures to class content to promote student engagement. (Kwok et al., 2023, p. 60)

Developing **data literacy** skills,

> “Within the research, deficit-based theories are pervasively used to explain Latine students’ challenges and experiences in STEM [...] one study attributed the disparate academic outcomes in calculus between White and Asian students and the lower scores of students of color to a lack of motivation, under-preparation, and lack of familial support [...] Without critical examination of the traditional ideologies, perspectives and biases that have shaped STEM pedagogies and practices, STEM educators can unknowingly employ and reproduce deficit-based interactions (lowered expectations, microaggressions) when working with Latine students” (Hernandez Negrete et al., 2023, p. 3).

> “Not only did I learn the importance of culturally relevant texts, but also giving students power in their own learning. [...] if you walked in my classroom, whether it was literacy or math, you could see me in the background and the kids doing the heavy lifting. My work in the critical inquiry group led to that, and that was a big change. Instead of teaching to a test, I teach students to understand lenses, perspective, and to become advocates.” (Friedman, 2024, p. 776)

**High-quality training** and **Structural support**,

> Thus, it should become the responsibility of teacher education programs (TEPs) and new teacher induction programs to combat this systemic phenomenon of victim blaming. By simply placing preservice teachers in urban field experiences, TEPs assume they are dismantling the deeply held stereotypes of young, White teachers (Holtz et al., 2024, p. 532)

> Regardless of the context, it was evident that participants experienced a positive paradigmatic shift in learning about anti-deficit perspectives on student success, which has important implications for practice (Harper, 2014). Graduate preparation programs as well as academic and student affairs units should consider exposing employees to this body of scholarship. [...] Centers for teaching on college campuses should also consider how anti-deficit perspectives can be integrated into orientation programs for new faculty. Otherwise, faculty are likely to continue replicating the same oppressive practices that create barriers to student success, especially among minoritized communities. (Pérez II et al., 2025, p. 650)

> “When we transform people and do not show them an avenue of support, we blame their lack of commitment rather than our lack of guidance.” (Kendi, 2023, pp 234-235)

And **ongoing reflective vigilance,**

> “In schools, leaders need a relentless focus on antiracist praxis which includes analyzing instructional efforts, professional development initiatives, and their own leadership through a lens that asks whether the work supports abolitionist teaching or not [...] For teacher education programs, this translates into infusing antiracist praxis into all coursework, pedagogy, and field work, rather than a single course focused on culture and race and otherwise allowing whiteness to remain unchecked” (Friedman, 2024, p. 780)

> The benefits of conducting a longitudinal study, as was this study, is that it revealed the importance of PSTs making intentional moves over time to make noticing in anti-deficit ways a more privileged and routine aspect of their instruction. Rather than emphasizing equitable instruction in one course or one assignment, anti-deficit noticing should be at the core of a teacher education program, supporting PSTs to move beyond conventional practices to view their students and teaching through an equitable lens. (Benedict-Chambers et al., 2025, p. 11)

## References for External Data Sources


1. Benedict-Chambers, A., Bolyard, C. & Belue, A. Anti-deficit framing and preservice teacher noticing for equity throughout a teacher education program. *Teaching and Teacher Education* **160**, 105007 (2025).
2. Cabiles, B. S. Internalised deficit perspectives: positionality in culturally responsive pedagogical frameworks. *Pedagogy, Culture & Society* **0**, 1–18 (2024).
3. Cushing, I. The sound of misbehaviour: deficit thinking and language policing in school discipline policies. *International Studies in Sociology of Education* **0**, 1–23 (2025).
4. Friedman, T. E. "The Students Led Me Here": A White Teacher's Movement Toward Antiracist and Abolitionist Practice. *Urban Rev* **56**, 763–783 (2024).
5. Harper, S. R. An anti-deficit achievement framework for research on students of color in STEM. *New Drctns for Instit Rsrch* **2010**, 63–74 (2010).
6. Hernandez Negrete, A., Mouavangsou, K. N. & Caporale, N. Toward asset-based LatCrit pedagogies in STEM: centering Latine students' strengths to reimagine STEM teaching and practice. *Front. Educ.* **8**, (2023).
7. Holtz, E., Worley, C. & Williams, J. A. Are Deficit Perspectives of Black and Brown Students Grounded in Empirical Data? Investigating the Myths of "Urban Education" Through Parent Satisfaction. *Education and Urban Society* **56**, 515–540 (2024).
8. Kendi, I. X. *How to Be an Antiracist*. (One World, New York, NY, 2023).
9. Kwok, M., Rios, A. & Kwok, A. Dispelling Deficit Framing: Investigating Preservice Teacher Beliefs About the Intersections between Literacy Instruction and Culturally Relevant Pedagogy. *Teachers and Teaching* **29**, 52–69 (2023).
10. Lowery, K. & Romero-Johnson, S. The Enactment of Anti-Deficit Thinking by Latina/o/x School Leaders. *Journal of Latinos and Education* **24**, 527–543 (2025).
11. Mampaey, J. & and Huisman, J. The reproduction of deficit thinking in times of contestation: the case of higher education. *British Journal of Sociology of Education* **43**, 1233–1249 (2022).
12. Pérez II, D. *et al.* Reimagining Student Success: Using Anti-Deficit Approaches to Educate Scholar-Practitioners in Higher Education. *Journal of Student Affairs Research and Practice* **61**, 639–652 (2024).
13. Valencia, R. R. *The Evolution of Deficit Thinking: Educational Thought and Practice.* (Routledge, Oxon, England, 1997).
14. Valencia, R. R. *Dismantling Contemporary Deficit Thinking*. (Routledge, New York, NY, 2010).
